# Efficacy of Adjunctive Local Antimicrobials to Non-Surgical Periodontal Therapy in Pocket Reduction and Glycemic Control of Patients with Type 2 Diabetes: A Network Meta-Analysis

**DOI:** 10.2174/0115733998320667240805045742

**Published:** 2024-09-03

**Authors:** Domitilla Marchiori Sant’Anna Leal de Oliveira, Ana Luiza Magalhães de Souza, Getúlio da Rocha Nogueira Filho, Carolina Castro Martins-Pfeifer, Cristine Miron Stefani

**Affiliations:** 1 School of Dentistry, University of Brasilia, Brasilia, Brazil;; 2 Private Practice, Dunedin, New Zealand;; 3 School of Dentistry, Federal University of Minas Gerais, Belo Horizonte, Brazil

**Keywords:** Antimicrobials, type 2 diabetes mellitus, periodontitis, non-surgical periodontal therapy, systematic review, network meta-analysis

## Abstract

**Objective:**

This network meta-analysis [NMA] investigated the efficacy of adjunctive use of subgingivally delivered antimicrobials to non-surgical periodontal therapy [NSPT] in the glycemic control and periodontal pocket depth (PPD) reduction in patients with type 2 diabetes (T2D).

**Methods:**

Seven databases, grey literature, and registry platforms were searched up to February 2024 to identify randomized clinical trials (RCT) fulfilling the eligibility criteria. The risk of bias was assessed through Cochrane’s tool (RoB 2). Two frequentist NMA were performed using a random-effects model to calculate mean differences (MD) as an effect measure and to quantitatively evaluate the glycated hemoglobin (HbA1c) and PPD. The certainty of evidence was assessed through the GRADE approach in a partially contextualized framework for interpreting results. Ten RCTs were included.

**Results:**

In total, 261 patients were treated with eight different local antimicrobials adjuvants to NSPT (azithromycin gel, clarithromycin gel, tetracycline fiber or ointment, chlorhexidine gel, doxycycline nanospheres, minocycline gel, and satranidazole gel), while 249 patients received NSPT alone or associated to placebo. Considering PPD reduction (8 included studies), the best results were found after six months for satranidazole gel (MD -2.64 mm; 95%CI -3.56, -1.72; moderate evidence certainty). For HbA1c control (7 included studies), doxycycline gel (MD -0.80%; 95%CI -1.70, 0.10), chlorhexidine gel (MD -0.68%; 95%CI -1.34, -0.02), and tetracycline fiber (MD -0.62%; 95%CI -0.85, -0.39) showed promising results after three months (low evidence certainty).

**Conclusion:**

The adjunctive use of satranidazole gel probably reduces PPD after a 6-month follow-up, while doxycycline gel, chlorhexidine gel, and tetracycline fiber may decrease HbA1c values in patients with T2D and periodontitis treated with NSPT after a 3-month follow up.

## 1 INTRODUCTION

Several studies have described the bidirectional relationship between periodontitis and T2D [1-3]. While diabetes increases the risk for periodontitis by 2-3 times and is associated with increased disease severity [4-6], periodontitis may be related to poorer glycemic control and a higher risk for diabetic complications [1, 4, 5]. Therefore, previous studies have investigated the effect of conventional periodontal treatment on diabetes control, showing a reduction in glycated hemoglobin (HbA1c) levels in patients with T2D and periodontitis [3, 6, 7].

The cornerstone of periodontitis treatment consists of mechanical debridement of the root surface, reducing clinical inflammation and periodontal pocket depth (PPD) [8-10]. This procedure, also known as non-surgical periodontal therapy (NSPT), includes mechanical removal of supra and subgingival bacterial plaque through scaling and root planing using scalers, curettes, or ultrasonic devices [7]. However, NSPT presents some limitations, such as difficulty accessing deep pockets and furcation areas [8, 9]. Therefore, in cases where NSPT alone cannot control periodontal disease, antimicrobial agents have been indicated to improve its results, especially in patients with systemic diseases that affect host resistance, as an alternative to surgical procedures [8, 11].

Antimicrobials include antibiotics and antiseptics that eliminate or inactivate pathogens in sites where NSPT is not entirely effective [11, 12]. These substances can be used locally or systemically as an adjunct to mechanical instrumentation [8, 11]. However, although systemic antibiotics may improve periodontal health, they should be indicated only for particular situations due to their systemic and microbiological side effects (including intestinal effects and toxicity) and the risk of resistant organisms rising [8, 9, 11]. On the other hand, local antimicrobials present fewer adverse effects, better compliance, and a lower chance of bacterial resistance [8], usually indicated in localized pockets or nonresponding and recurrent sites [8,9]. Besides, the local (subgingivally delivered) route can achieve antimicrobial concentration levels that the systemic route cannot reach and allow antiseptic substances that would be toxic if systemically administered [11].

Literature has shown the benefits of subgingivally delivered antimicrobials used as adjuncts to NSPT in periodontitis treatment of normoglycemic patients [9, 12, 13] and patients with T2D [14]. The adjunctive use of these substances may improve periodontal parameters in systemically healthy patients, reducing pathogenic bacterial load in biofilm and proinflammatory mediators release, which in diabetes could help control HbA1c levels [15, 16]. Furthermore, it is noteworthy that HbA1c control in patients with T2D requires a previous periodontal control [17], and these patients have been considered poor candidates for surgical procedures due to their impaired healing [18, 19]. So, adjunctive therapies can be beneficial to this population [20].

A previous systematic review evaluated the efficacy of local antibiotics in patients with either type 1 or type 2 diabetes and showed periodontal improvement, with PPD reduction and clinical attachment level (CAL) gain, when these substances were associated with NSPT, especially in deep sites [14]. However, studies with antiseptic agents, such as chlorhexidine, were not included. In addition, the effects of local antibiotics on glycemic status and the certainty of evidence were not assessed. So, these results must be revisited.

Thus, this systematic review with network meta-analysis aimed to evaluate the effectiveness of subgingivally delivered local antimicrobials (including antibiotics and antiseptics) associated with NSPT in the glycemic control and periodontal parameters in patients with T2D and periodontitis.

## 2 MATERIAL AND METHODS

### 2.1 Protocol and Registration

The study protocol of this network meta-analysis was registered in The International Prospective Register of Systematic Reviews – PROSPERO (registration number CRD42022308714), available at http://www.crd.york.ac.uk/prospero/, and the review adhered to the PRISMA Extension Statement for Reporting of Systematic Reviews Incorporating Network Meta-analyses of Health Care Interventions [21].

### 2.2 Focused Question

The following focused question was developed according to the PICO acronym (Population, Intervention, Comparison, and Outcomes): “In patients with type 2 diabetes and periodontitis (P), does the adjunctive use of subgingivally delivered local antimicrobials to non-surgical periodontal therapy (I) improve the glycemic control and clinical periodontal parameters (O) when compared to conventional non-surgical periodontal therapy alone or associated to placebo (C)?

### 2.3 Information Sources and Search Strategy

An electronic search was conducted on February 04, 2022, and updated on February 25, 2024, in PubMed (MEDLINE), Cochrane (CENTRAL), Embase, LIVIVO, Scopus, Web of Science, and LILACS databases. Grey literature (non-peer-reviewed literature) was also searched through ProQuest (Dissertation and Theses), OpenGrey, and Google Scholar. The Clinical Trials Registry (available at https://clinicaltrials.gov/) and the International Clinical Trial Registry Platform (ICTRP WHO, available at https://trialsearch.who.int/) were also analyzed, besides the manual searches of the reference lists from selected studies. All searches were done without language or date restrictions, and the main descriptors were defined as antimicrobials, T2D, and non-surgical periodontal therapy.

On the PubMed database, the Medical Subject Headings [MeSH] Terms searched were: (“Antimicrobial”) AND (“Diabetes Mellitus” OR “Glycated Hemoglobin A”) AND (“Chronic Periodontitis” OR “Periodontitis” OR “periodontal debridement”), including words variations and synonyms. Supplementary file **S1** presents the complete search strategy for each database searched.

### 2.4 Eligibility Criteria

The PICO acronym was used to define the inclusion and exclusion criteria. Inclusion criteria consisted of randomized controlled clinical trials (RCT) evaluating (P) adult patients with T2D with untreated periodontitis who underwent (I) NSPT associated with subgingivally delivered local antimicrobials [including antibiotics and antiseptics] compared to (C) control groups (NSPT alone or combined to placebo). Glycemic control and clinical periodontal parameters, evaluated respectively through HbA1c and PPD level changes from baseline and at least 2-month follow-up, were considered the primary outcomes (O). The Periodontitis case definition was determined according to the new Periodontal Diseases Classification [22], and the diabetes case definition according to the International Expert Committee [23]: HbA1c ≥6.5%.

We excluded studies with non-RCT design, studies with no full text available, studies involving patients with type I diabetes, studies evaluating T2D children and adolescents, studies in which subgingivally delivered local antibiotics/antimicrobials were combined with other active therapy [systemic antibiotics, anti-inflammatory drugs, *etc*.), studies not evaluating HbA1c or PPD as outcomes, studies using local antimicrobials/antibiotics with no low liberation vehicles use (*e.g.*, with fluids or aqueous solutions as vehicles), and studies using non-absorbable delivery devices (*e.g.*, plastic tubules).

### 2.5 Study Selection

We imported all electronic search results to the reference manager Mendeley Desktop software (v.1.19.8, Elsevier, Amsterdam, The Netherlands), and excluded the duplicated records. Study selection was performed in the Rayyan QCRI online application [24] in two phases. In phase 1, two reviewers (DMSLO and ALMS) independently evaluated titles and abstracts following the previously established eligibility criteria, and a third reviewer (CMS) evaluated disagreements. Phase 2 was also carried out independently by the same two reviewers evaluating the study's full texts to confirm eligibility. Once more, the same third evaluator solved divergences.

### 2.6 Data Extraction

Two reviewers (DMSLO and ALMS) extracted data independently with posterior comparison and contacted the authors of the included studies to provide missing data if necessary. The following variables were extracted to summarize descriptive characteristics of included articles: 1) authors, publication year, and country; 2) age in years or mean age of participants; 3) definitions of diabetes and periodontitis reported by the authors; 4) sample size; 5) information about antimicrobial substance tested; 6) results regarding HbA1c and/or PPD before and after treatment for test and control groups; 7) follow up; 8) main conclusions reported in the included study; and 9) information about conflict of interest and funding sources.

If the results of any included study were presented only graphically (no quantitative data on the mean and standard deviations for HbA1c levels), the data for HbA1c for each group were extracted from the published plot using WebPlotDigitizer online application (v4.5, Ankit Rohatgi, Pacifica, CA, USA), and the standard deviations were calculated using an online app (https://www.calculator.net/standard-deviation-calculator.html, assessed on April 21, 2022).

### 2.7 Risk of Bias in Individual Studies

The quality assessment of the included studies was performed by the same two reviewers (DMSLO and ALMS) according to Cochrane’s risk of bias tool for RCT (RoB 2) [25], considering the “per protocol” analysis for each outcome (HbA1c and PPD). PPD was the chosen periodontal outcome because it was a standard clinical periodontal parameter found in all included studies. Besides, it is considered a core outcome in clinical studies in periodontics [26]. Each included study was assessed individually for five predefined domains (risk of bias arising from the randomization process, risk of bias due to deviations from intended interventions, missing outcome data, risk of bias in measurement of the outcome, and selection of the reported results). Based on the answers, the domain was classified by the tool as “low risk”, “some concerns”, or “high risk” of bias. The worst classification among the domains was responsible for the overall risk of bias in the study. Once again, a third evaluator (CMS) checked for disagreements.

### 2.8 Data Synthesis and Statistical Analysis

For every included study, we calculated mean scores (and standard deviations) regarding HbA1c and PPD level change from baseline to 12 and 24 weeks (3 and 6 months) after treatment. After that, using the MetaInsight online software V3.1.14 [27] for continuous variables [available on https://crsu.shinyapps.io/MetaInsight], we performed a frequentist network meta-analysis [NMA] to compare different local antimicrobials used as an adjuvant treatment to NSPT. We calculated mean differences [MD] with a 95% confidence interval using the random effects method model and inverse variance statistics. Diagrams were used to analyze the geometry of the network.

### 2.9 Certainty of Evidence Assessment

Two reviewers [DMSLO and CMS] evaluated the certainty of evidence according to the GRADE approach with a Partially Contextualized Framework for Network Meta-analysis to interpret results [28, 29], a similar methodology used in other studies with NMA [30, 31]. To evaluate the quality of direct comparisons, we assessed the risk of bias, inconsistency, indirectness, and publication bias. For indirect comparisons, first-order loop comparison with the lowest certainty was considered, and intransitivity was assessed. Incoherence could not be evaluated due to the poorly connected network, but imprecision was judged for the NMA effect estimate. Supplementary Tables **S1**-**S4** present detailed information on judgment criteria mention in Supplementary file **S5**.

The partially contextualized framework considers the magnitude of the effect and the certainty of the evidence for interpreting the NMA results [32]. The magnitude of the effect followed the Minimal Important Difference (MID). The MID was the MD between groups, interpreted as the smallest beneficial difference that would mandate a therapy without troublesome adverse effects and excessive costs [33]. For HbA1c, a reduction of 0.5% was considered the MID; for PPD, the MID was a periodontal pocket reduction of ≥ 1 mm, or the change necessary to distinguish a periodontally diseased site from a healthy one [*i.e.*, from 4mm to 3mm]. Both MID values were decided by discussion within the review team.

## 3 RESULTS

### 3.1 Study Selection

The initial literature search returned 1,819 records, and then 586 duplicates were excluded, with the remaining 1,233 studies for phase 1 of the study screening process. Titles and abstracts from the 1,233 records were analyzed according to the eligibility criteria, and 1,206 were removed, resulting in 27 articles for phase 2. All twenty-seven full texts were evaluated, and seventeen were deleted, resulting in 10 included studies.

**Fig. (1):**
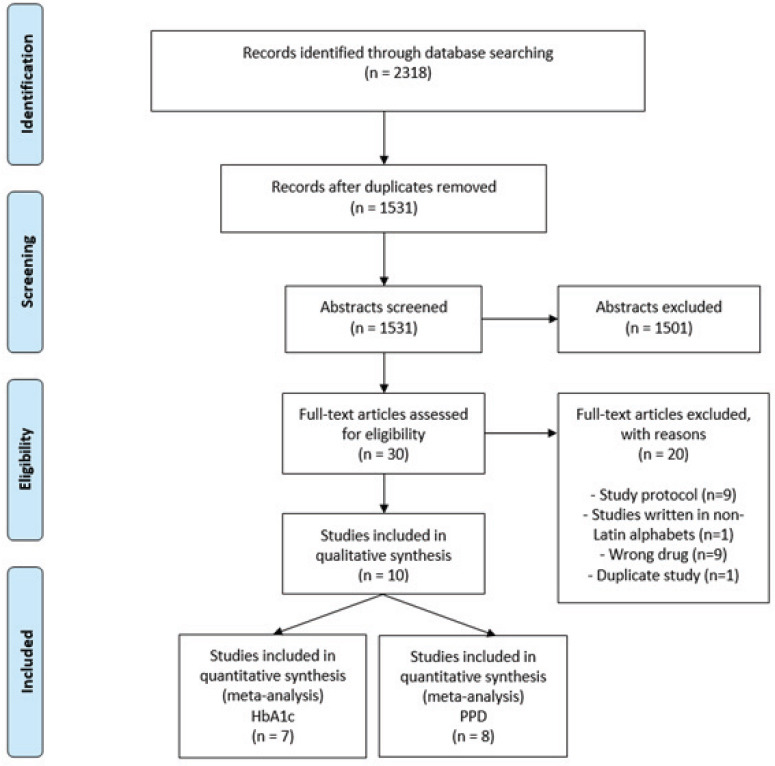
Flow diagram of the selection process according to the PRISMA statement for NMA.

The search update returned 298 new records, and 295 were excluded after title and abstract reading. The full texts of three studies were evaluated, and none was included, with the remaining 10 studies included in the first search (Fig. **1**). Supplementary file **S2** presents a list of excluded articles and reasons for exclusion.

### 3.2 Included Studies

The characteristics of the included studies are shown in Table **1**. The studies were published between 2009 and 2019 in India [34-37], Iran [38], Brazil [39, 40], Taiwan [41], and Japan [42, 43]. All authors reported a clinical periodontal examination and laboratory tests for HbA1c level to confirm the diagnoses of periodontitis and T2D, respectively. In test groups (TG), patients were treated with subgingivally delivered antimicrobials adjuvant to NSPT, while in control groups (CG), subjects underwent NSPT alone [36, 38, 41-43] or associated with a placebo [34, 35, 37, 39, 40].

In total, 7 studies evaluated HbA1c [36, 38-43], 9 PPD [34–42], and 6 outcomes [36, 38-42]. Considering all test groups, 31 patients were treated with 0.5% azithromycin gel [34] injected into periodontal pockets after NSPT (maximum PPD, 1 site per patient); 32 with 0.5% clarithromycin gel [35] also injected into periodontal pockets after NSPT [maximum PPD, 1 site per patient]; 20 with resorbable tetracycline fiber [36] inserted into selected periodontal pockets after NSPT and covered by a periodontal pack for 10 days; 34 with 1.5% chlorhexidine gel [38] inserted into periodontal pockets (8 teeth with 4-8 mm PPD, minimum) after two sessions of NSPT (2 weeks apart) and covered by a periodontal pack for 7 days; 23 with 20% doxycycline-loaded PLGA nanospheres [39] applied into periodontal pockets (4 moderate and 2 deep pockets) after NSPT; 14 with 2% minocycline gel [41] inserted into periodontal pockets in a 4-week regimen, one month after NSPT; 14 with 2% minocycline gel [42] applied into periodontal pockets (every site with PPD ≥4 mm), after NSPT; 42 with tetracycline HCl ointment [43] applied into every periodontal pocket (once a week for 4 weeks) combined with sessions of NSPT; 32 with 3% satranidazole gel [37] injected into periodontal pockets after NSPT; and 19 with 1% chlorhexidine gel [40] applied into periodontal pockets, three times (repeated within 10 minutes), after two appointments of NSPT within 24h. In control groups, 114 patients underwent NSPT alone [36, 38, 41-43], while 135 received NSPT associated with a placebo [34, 35, 37, 39, 40].

Munenaga *et al*. (2013) [43] included six groups in the study, but according to the periodontitis case definition, only two groups (one test and one control) were analyzed in the NMA.

### 3.3 Risk of Bias

**Fig. (2):**
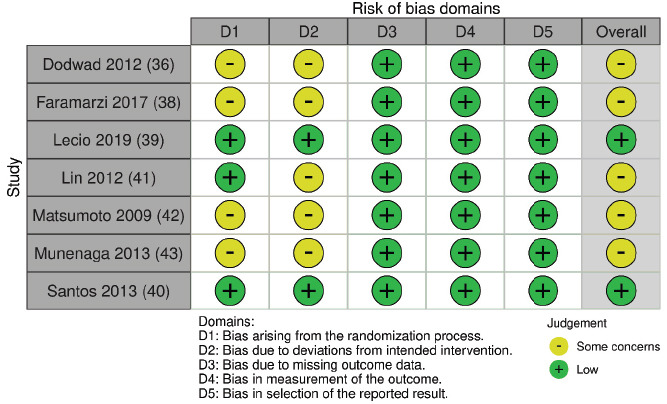
Risk of bias assessment of included studies (for HbA1c outcome) considering each Cochrane’s RoB 2 tool domain and overall risk of bias.

**Fig. (3):**
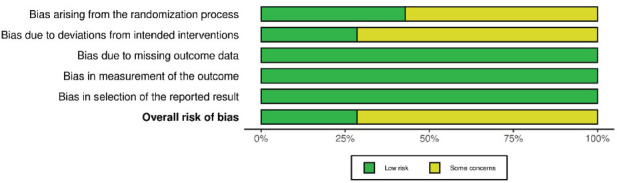
Weighted bars plot of Risk of bias evaluation of included studies (for HbA1c outcome) considering Cochrane’s RoB 2 tool.

Regarding HbA1c, two studies [39, 40] presented a “low” risk of bias, and five studies presented [36, 38, 41-43] “some concerns” (Figs. **2** and **3**). The risk of bias in the studies classified as “some concerns” was due to poor description of the randomization process and allocation concealment and the fact that the operators were not blinded.

**Fig. (4):**
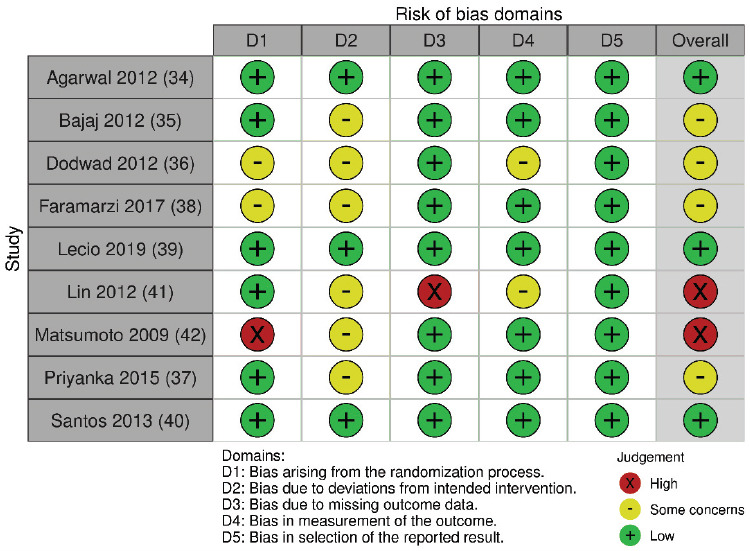
Risk of bias assessment of included studies (for PPD outcome) considering each Cochrane’s RoB 2 tool domain and overall risk of bias.

**Fig. (5):**
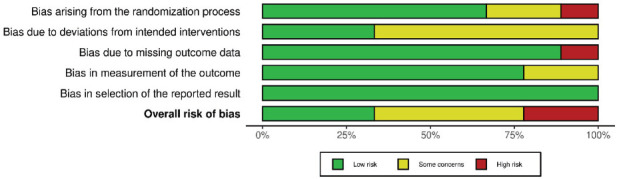
Weighted bars plot of Risk of bias evaluation of included studies (for PPD outcome) considering Cochrane’s RoB 2 tool.

Considering PPD, three studies [34, 39, 40] presented a “low” risk of bias, four studies [35–38] were considered with “some concerns”, and two studies [41,42] had a “high” risk of bias (Figs. **4** and **5**). The main reasons for the increased risk of bias were lack of information about the randomization process, allocation concealment, or the lack of operator or outcome assessor blinding. Furthermore, one study [41] did not provide missing data outcome about PPD.

We emphasize that in the study performed by Matsumoto *et al*. (2009) [42], test and control groups presented statistical differences concerning PPD at baseline but not regarding HbA1c, which justifies the different classifications in domain 1 (randomization process), according to Cochrane’s RoB 2 algorithm.

### 3.4 Network Meta-analysis Results

Seven studies were included in the NMA for HbA1c, while eight were for PPD. The study performed by Matsumoto *et al*. (2009) [42] was not included in the meta-analysis for PPD because the authors mentioned the improvement in PPD after using minocycline gel adjunct to NSPT, but mean±SD was not available either at baseline or after the treatment, and further information was not obtained after contact with the authors. Fig. (**6**) (HbA1c) and Fig. (**7**) (PPD) show the network plot with the number of interventions and studies included for each outcome (HbA1c and PPD) at different follow-up periods (3 and 6 months). Supplementary file **S3** presents the league table for all effect estimate comparisons.

Tables **2** and **3** show the effect estimates for each comparison, considering the outcomes and different follow-up periods (3 and 6 months). The results were interpreted using the GRADE partially contextualized framework.

At 3 months, doxycycline nanospheres (MD: -0.80%; 95%CI: -1.70, 0.10), chlorhexidine gel (MD: -0.68%; 95%CI: -1.34, -0.02), and tetracycline fiber (MD: -0.62%; 95%CI: -0.85, -0.39) reached the MID in HbA1c reduction when compared to NSPT, all with low certainty of evidence. At 6 months, only the chlorhexidine gel (MD: -0.53%; 95%CI: -1.57, 0.51) was more effective than NSPT, achieving the MID, yet with very low certainty of evidence (Table **2**).

At 3 months, satranidazole gel (MD: -1.30 mm; 95%CI: -2.22, -0.38) and clarithromycin gel (MD: -1.01 mm; 95%CI: -1.50, -0.52) reached the MID in PPD reduction, when compared to NSPT (low certainty of the evidence). The best result was found for satranidazole gel after 6 months, which reduced PPD by 2.64 mm (about 2.5x the MID) compared to NSPT with moderate certainty of evidence (MD: -2.64 mm; 95%CI: -3.56, -1.72) (Table **3**).

Fig. (**8**) (HbA1c) and Fig (**9**) [PPD] present, in forest plots, a comparison of all local antimicrobials and NSPT alone. Supplementary files **S4**-**5** show inconsistent test results.

### 3.5 Certainty of Evidence

At 3 and 6 months, the certainty of the evidence for HbA1c varied from “low” to “very low,” while for PPD, from “moderate” to “very low” (Supplementary file **S3**). The risk of bias and imprecision were the main reasons for the reduction of the level of evidence. Supplementary file **S5** presents detailed results for each comparison judgment.

## 4 DISCUSSION

The results of the NMA were analyzed, considering the magnitude of the effect and the certainty of the evidence. The most promising subgingivally delivered local antimicrobials (used as adjuncts to NSPT and compared to NSPT alone) for HbA1c reduction after 3 months were doxycycline gel, chlorhexidine gel, and tetracycline fibers. All of them reached the MID for HbA1c, nevertheless, all with low evidence certainty. After 6 months, chlorhexidine gel showed promising effects despite the very low certainty.

Concerning PPD, satranidazole gel was the most effective treatment compared to NSPT after a 6-month follow-up (large effect with moderate certainty).

Satranidazole is a novel nitroimidazole, an alternative to metronidazole, presenting four-fold more potency against gram-negative bacteria [44]. This substance has been considered more active against aerobic, anaerobic, and microaerophilic bacteria, being an interesting alternative to eliminate deep pockets when combined with NSPT. Satranidazole can potentially reduce sites infected by periodontopathogens, such as *Porphyromonas gingivalis, Tannerella forsythia*, and *Aggregatibacter actinomycetemcomitans* [45]. The subgingival use of 3% satranidazole gel in systemically healthy subjects with periodontitis showed a more significant PPD reduction and CAL gain than NSPT alone and metronidazole gel [45,46]. A more recent study compared the efficacy of 0.25% satranidazole gel *versus* 1% metronidazole gel subgingival use in association with NSPT in the treatment of periodontitis, showing that even at lower concentrations, satranidazole gel presented good results [44]. The literature is still scarce in evaluating the benefits of local satranidazole in diabetic patients, and our search retrieved only one study [37], which was considered with “some concerns” regarding the risk of bias due to the lack of operator blinding. Although the NMA showed a large effect with moderate certainty for PPD reduction after 6 months of follow-up, the effect of satranidazole gel on HbA1c was not tested in this study. Thus, although promising, further studies are necessary to interlink the periodontal results to glycemic control and confirm the effects on the periodontal parameters of patients with T2D.

Doxycycline belongs to the tetracycline family and is a broad-spectrum antibiotic with additional benefits like anti-oxidant properties and the capacity to inhibit matrix metalloproteinases (MMPs) that induce the release of various inflammatory mediators [47, 48]. There are studies demonstrating the benefits of local doxycycline for improving periodontal status in systemically healthy subjects [49-52], patients with type 1 diabetes [53], and smokers [54]. In the same way, some studies have already described an HbA1c reduction [48,55,56] and periodontal improvement [48,56] in patients with T2D, but with the systemic use of the drug. Gomaa *et al*. (2018) [57] treated T2D patients with systemic doxycycline 20 mg and subgingivally delivered doxycycline gel 10% into the periodontal pockets. The patients exhibited a more significant reduction of PPD and CAL [*P<*0.0^5^] when compared to the control group (NSPT alone) [57]. However, this study was excluded from the present review due to the concomitant use of systemic antibiotics. Our search returned only one study evaluating the local use of doxycycline in patients with T2D and periodontitis [39], and it presented a “low” risk of bias. However, the NMA showed an important effect with low certainty, so these findings need further confirmation.

Chlorhexidine is an effective antiseptic against a broad spectrum of microorganisms. When administered in gel-based presentations, higher concentrations (up to 15 times) are reached than liquid carriers [58]. Chlorhexidine gel as an adjuvant to NSPT can improve the periodontal status, reducing PPD and increasing CAL gain in patients with periodontitis [58-60]. In the study by Srirangarajan *et al*. (2016) [59], patients with T2D, with and without periodontitis, were treated with NSPT in a comparative clinical trial. Those with periodontitis also received subgingivally delivered chlorhexidine 1% gel in all periodontal pockets. After the treatment, all periodontal parameters (plaque index, gingival index, and PPD) significantly improved. All patients presented a statistically significant reduction in fasting glucose, suggesting that decreasing periodontal inflammation reduces insulin resistance, improving metabolic control [59]. Two studies evaluating the efficacy of chlorhexidine were included in the present network meta-analysis [38, 40]. One [40] considered a “low” risk of bias, and the other one [38] presented “some concerns”. The NMA showed an important and consistent effect for CHX gel on HbA1c reduction after 3 and 6-month follow-up; however, with low certainty of evidence after 3 months and very low after 6 months. Therefore, these results must be analyzed cautiously due to considerable uncertainty and demand confirmation with further studies.

Tetracyclines are a common antibiotic family with antimicrobial, anti-inflammatory, and anti-collagenase properties, besides the ability to inhibit bone resorption and promote the attachment of fibroblasts to root surfaces [47, 61]. Tetracyclines are bacteriostatic agents effective against many gram-negative species, such as *A. actinomycetemcomitans*. Due to their antimicrobial and immunomodulatory effects, tetracyclines are a good adjunctive option in managing periodontal disease [47, 61]. The effects of tetracycline fibers have been studied since 1983, indicating that these devices could affect the periodontal microbiota and signs of clinical improvement [62]. Some studies tested the efficacy of subgingivally inserted tetracycline fiber as an adjunct to NSPT compared to NSPT alone and showed a greater PPD reduction and CAL gain [63-65]. Other methods for the subgingival delivery of tetracyclines include irrigation and the subgingival application of an ointment or gel [66]. There is evidence that the delivery through fibers is more efficient than irrigation [67], although there is no comparison with other formulations (gel or ointment). We included only one study using tetracycline fiber in patients with T2D [36] and one with tetracycline ointment [43]. The tetracycline ointment showed a slight reduction in HbA1c with very low certainty. In contrast, the tetracycline fiber showed an important effect, with low certainty for the HbA1c decrease after 3 months follow-up. Furthermore, no additional studies were found to reinforce these findings. Therefore, these results require further confirmation.

It is noteworthy that the effectiveness of each antimicrobial is not only based on the drug tested but also on the characteristics of the patient as well as the infection. Many factors may interfere with the efficacy of antimicrobials, such as dosage, vehicle, duration of treatment, broadness of spectrum, and timing of therapy [68]. Additionally, the treatment of T2D includes lifestyle changes, dietary changes, and weight loss, as well as pharmacological therapy [4]. All these factors can help explain why some antimicrobials present better results than others.

Periodontitis control favors glycemic control and decreases systemic inflammation in patients with T2D [69]. Chronic infections, including periodontitis, might increase circulating cytokines and factors, such as C-reactive protein, interleukin-1 beta, interleukin-6, tumor necrosis factor-alpha, and prostaglandin-E2 [70]. As a result, the systemic inflammatory burden increases, which eventually induces insulin resistance in T2D, impairing glycemic control [7, 70]. Patients with T2D and periodontitis have higher levels of systemic inflammatory markers compared with people with periodontitis only, and NSPT results in a greater reduction of these markers in subjects with T2D compared with those with periodontitis and systemically healthy [71]. In this context, periodontal therapy may improve insulin sensitivity and, consequently, the HbA1c values by decreasing inflammatory cytokines and markers levels [69, 71]. Since any improvement in glycemic status has the potential to reduce the risk of diabetic complications and to improve the quality of life of people with T2D [71, 72], any effort to control periodontal inflammation is valuable. Therefore, subgingival antimicrobials as adjuncts to NSPT in patients with T2D and periodontitis should be further investigated.

## CONCLUSION

The subgingival (local) use of satranidazole gel probably resulted in PPD reduction after a 6-month follow-up compared to NSPT alone (moderate evidence certainty) in patients with T2D; however, its effect on HbA1c has not been tested yet. Doxycycline gel, chlorhexidine gel, and tetracycline fiber as adjuncts to NSPT may reduce the HbA1c values in patients with T2D (low evidence certainty) after 3 months of follow-up, but these short-term findings require further confirmation.

## LIMITATIONS AND STRENGTHS

The primary limitation of this paper is the small number of included studies, which resulted in a poorly connected network. Furthermore, only some of the studies evaluated both clinical periodontal parameters and HbA1c value, preventing the inclusion of the ten included studies in both NMA (HbA1c and PPD).

Despite not being an objective of this study, clinicians are interested in knowing the adverse effects of the tested substances. However, none of the studies evaluating the local antimicrobials with promising results reported these data.

On the other hand, the strengths of this study include the highly methodological rigor used for assessing the certainty of evidence and interpreting the results through the GRADE partially contextualized framework with MID, which can prevent mistaken conclusions [32].

### IMPLICATIONS FOR FUTURE RESEARCH AND CLINICAL PRACTICE

The effectiveness of adjunctive local antimicrobials to NSPT on reducing both HbA1c levels and improving clinical periodontal parameters (PPD) in patients with T2D and periodontitis is still unclear. So, further intervention trials are required to confirm these NMA findings. These might include larger sample sizes, longer follow-ups, and rigorous methods to reduce the risk of bias and increase the validity of the results. Besides that, we recommend that future research focus on the antimicrobials that achieved MID, although with low and very low certainty, as future research may change the current evidence [73]. The antimicrobials with promising results that should be included in future trials are satranidazole, doxycycline, chlorhexidine, tetracycline, and clarithromycin.

## Figures and Tables

**Fig. (6):**
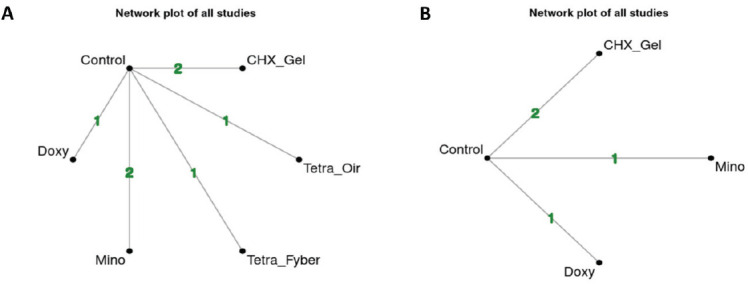
Network plot for included studies for HbA1c. Numbers in lines show the number of included studies in direct comparisons (CHX_Gel: chlorhexidine gel; Doxy: doxycycline gel; Mino: minocycline gel; Tetra_Fyber: tetracycline fiber; Tetra_Oin: tetracycline ointment). (**A**): network plot at 3 months; (**B**): network plot at 6 months.

**Fig. (7):**
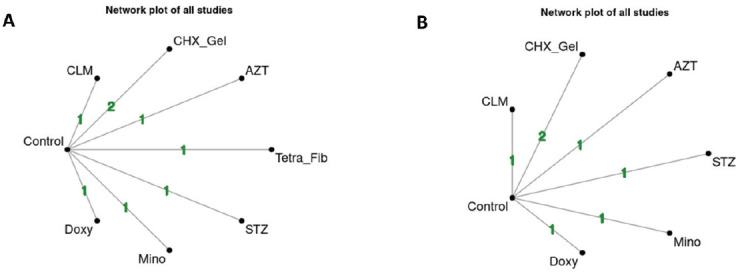
Network plot for included studies for PPD. Numbers in lines show the number of included studies in direct comparisons (AZT: azithromycin gel; CLM: clarithromycin gel; CHX_Gel: chlorhexidine gel; Doxy: doxycycline gel; Mino: minocycline gel; Tetra_Fib: tetracycline fiber; STZ: satranidazole gel). (**A**): network plot at 3 months; (**B**): network plot at 6 months.

**Fig. (8):**
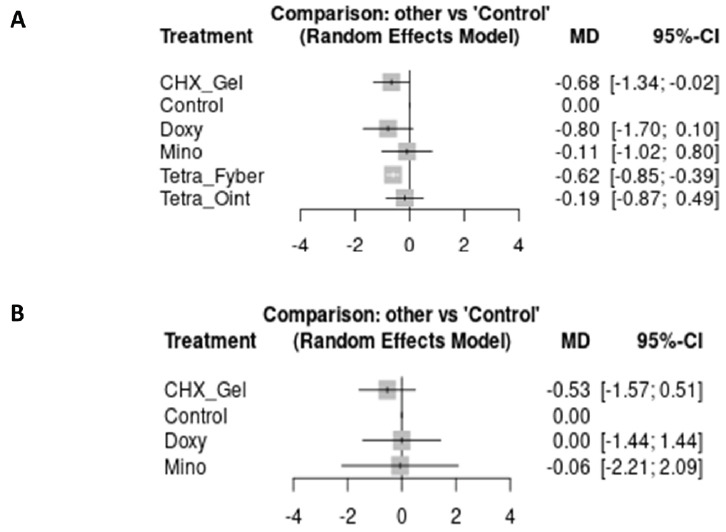
Forest plot showing the performance of different local antimicrobials used as adjuncts to NSPT, compared to NSPT alone after different follow-up periods. (**A**): HbA1c assessment at 3 months; (**B**): HbA1c assessment at 6 months.

**Fig. (9):**
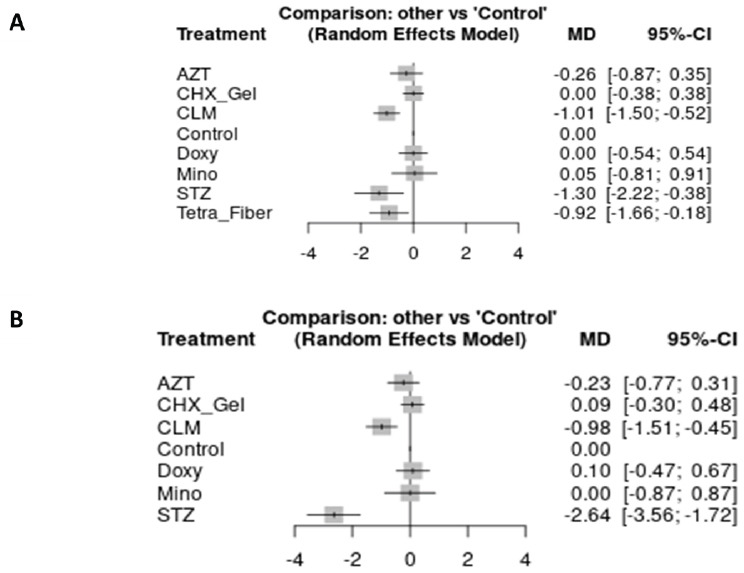
Forest plot showing the performance of different local antimicrobials used as adjuncts to NSPT, compared to NSPT alone after different follow-up periods. (**A**): PPD assessment at 3 months; (**B**): PPD assessment at 6 months.

**Table 1:** Summary of descriptive characteristics of included articles (n=10).

Author, Year Country	Age in Years Mean ±SD or Range)	Case Definitions	Groups (n)	Treatments TG CG	Outcomes Comparison between Groups (Baseline) Mean ± SD	Follow Up (in Months)	Outcomes Comparison between Groups (Final / *P* value) Mean ± SD	Main Conclusions Reported in the Included Study	Conflict of Interest and Funding Sources
Agarwal *et al*., 2012 India	Age range 30-50 years	Periodontitis: PPD ≥5 mm and/or CAL≥4 mm and vertical bone loss≥3mm on intraoral periapical radiographs Diabetes: HbA1c≥6.5% and FPG≥126 mg/dl	TG (n=31) CG (n=32)	TG: NSPT + 0.5% azithromycin gel injected into periodontal pockets after NSPT (maximum PPD, 1 site per patient) CG: NSPT + placebo	PPD (mm) TG 7.38±0.71 CG 7.27±0.73	3 months 6 months 9 months	PPD (mm) TG 5.88±0.94 (*P<*0.05) CG 6.03±0.90 (*P<*0.05) CBG: NS PPD (mm) TG 4.98±0.50 (*P<*0.05) CG 5.10±0.75 (*P<*0.05) CBG: (*P<*0.05) PPD (mm) TG 3.96±0.57 (*P<*0.05) CG 4.51±1.25 (*P<*0.05) CBG: (*P<*0.05)	Local delivery of 0.5% azithromycin gel into periodontal pockets of CP patients with T2D stimulated a significant improvement in clinical improvement compared to placebo gel as an adjunct to NSPT.	No conflict of interest. Sources of support: Micro Labs (pharmaceutical company), Bangalore, India, and Purac Biomaterials (PURASORB), Gorinchem, the Netherlands.
Bajaj *et al*., 2012 India	Age range 30-50 years	Periodontitis: PPD ≥5 mm and/or CAL≥4 mm and vertical bone loss≥3 mm on intraoral periapical radiographs Diabetes: HbA1c≥6.5% and FPG≥126 mg/dl	TG (*n=*32) CG (*n=*31)	TG: NSPT + 0.5% clarithromycin gel injected into periodontal pockets after NSPT (maximum PPD, 1 site per patient) CG: NSPT + placebo	PPD (mm) TG 7.29±0.58 CG 7.23±0.69	3 months 6 months	PPD (mm) TG 4.93±0.39 NS CG 5.88±0.84 NS CBG: (*P<*0.05) PPD (mm) TG 5.14±0.40 (*P<*0.05) CG 6.06±0.88 NS CBG: (*P<*0.05)	Local delivery of 0.5% clarithromycin gel into periodontal pockets of T2D patients led to a significant reduction in PPD and gain in CAL as compared to placebo gel as an adjunct to NSPT.	Conflict of interest: not informed. Sources of support: Micro Labs (pharmaceutical company), Bangalore, India and Purac Biomaterials (PURASORB), Gorinchem, the Netherland.
Dodwad *et al*., 2012 India	Age range 35-70 years	Periodontitis: At least 3 sites with PPD ≥4 mm and ≤7 mm Diabetes: controlled or moderately controlled T2D	TG (*n=*20) CG (*n=*20)	TG: NSPT + Resorbable tetracycline fiber inserted into selected periodontal pockets after NSPT (periodontal pack for 10 days) CG: NSPT alone	HbA1c (%) TG 7.56±0.23 CG 7.65±0.23 PPD (mm) TG 3.55±0.81 CG 3.27±0.88	3 months	HbA1c (%) TG 6.87±0.34 *P=*0.000 CG 7.58±0.23 *P=*0.025 CBG: (*P=*0.000) PPD (mm) TG 2.14±0.54 *P=*0.000 CG 2.78±0.96 *P=*0.000 CBG: (*P=*0.015)	Locally delivered tetracycline is a better treatment modality compared to NSPT alone.	Conflict of Interest: None declared. Source of Support: Nil.
Faramarzi *et al*, 2017 Iran	TG 52.7 ± 7.3 CG 55.3 ± 8.8	Periodontitis: At least 20 teeth; minimum of 8 teeth with PPD ≥5 mm Diabetes: HbA1c≥6%	TG (*n=*34) CG (*n=*34)	TG: NSPT + subgingival 1.5% xanthan-based chlorhexidine gel inserted into periodontal pockets (8 teeth with 4-8 mm PPD, minimum) after two sessions of NSPT (2 weeks apart) (periodontal pack for 7 days) CG: NSPT alone	HbA1c (%) TG 7.72±0.99 CG 7.32±1.06 PPD (mm) TG 5.41±0.80 CG 5.41±0.76	3 months 6 months	HbA1c (%) TG 6.20±0.97 (*P<*0.05) CG 6.53±1.06 (*P<*0.05) CBG: NS PPD (mm) TG 3.48±0.56 (*P<*0.05) CG 3.67±0.83 (*P<*0.05) CBG: NS HbA1c (%) TG 6.06±1.04 (*P<*0.05) CG 6.42±1.02 (*P<*0.05) CBG: NS PPD (mm) TG 3.38±0.55 (*P<*0.05) CG 3.48±0.67 (*P<*0.05) CBG: NS	The application of chlorhexidine gel might improve the effects of NSPT in T2D patients with periodontitis.	No conflicts of interest. Financial support and sponsorship: Nil.
Lecio *et al*, 2019 Brazil	TG 58.6±12.4 CG 53.1±10.2	Periodontitis: At least 8 single-rooted teeth with PPD ≥5 and BoP Diabetes: History T2D> 5 years	TG (*n=*23) CG (*n=*21)	TG: NSPT + 20% doxycycline-loaded PLGA nanospheres applied into periodontal pockets (4 moderate and 2 deep pockets) after NSPT CG: NSPT + placebo nanospheres	HbA1c (%) TG 8.1±1.0 CG 8.4±1.2 PPD (mm) TG 5.6±0.5 CG 5.5±0.5	3 months 6 months	HbA1c (%) TG 6.9±1.2 (*P≤*0.05) CG 8.0±0.9 NS CBG: (*P≤*0.05) PPD (mm) TG 3.5±0.7 (*P≤*0.05) CG 3.4±0.7 (*P≤*0.05) CBG: NS HbA1c (%) TG 8.2±1.5 NS CG 8.5±1.6 NS CBG: NS PPD (mm) TG 3.3±0.7 (*P≤*0.05) CG 3.1±0.7 (*P≤*0.05) CBG: NS	DOXY nanospheres may be considered a potential adjunct to NSPT in the therapy of T2D.	No conflicts of interest. Funding: support from Research Support Foundation of the State of São Paulo – FAPESP.
Lin *et al*., 2012 Taiwan	TG 56.6±7.8 CG 59.0±6.5	Periodontitis: At least 20 teeth and five or more teeth with PPD≥5mm Diabetes: HbA1c≥8.5% for more than 5 years	TG (*n=*14) CG (*n=*14)	TG: NSPT + 2% minocycline gel inserted into periodontal pockets (4-week regimen) one month after NSPT CG: NSPT alone	HbA1c (%) TG 9.3±0.8 CG 9.9±2.2 PPD (mm) TG 5.13±0.47 CG 5.15±0.52	3 months 6 months	HbA1c (%) TG 9.11±1.13 NS CG 9.83±1.42 NS CBG: NS PPD (mm) TG 3.39±0.91 (*P<*0.05) CG 3.36±1.12 (*P<*0.05) CBG NS HbA1c (%) TG 8.09±2.60 NS CG 8.75±1.31 NS CBG: NS PPD (mm) TG 3.11±0.92 (*P<*0.05) CG 3.13±1.10 (*P<*0.05) CBG NS	NSPT alone or associated with minocycline may improve glycemic control in patients with poorly controlled T2D.	No conflicts of interest. Supported by funds SKH-TMU-96-12 from grants of cooperative projects of Shin-Kong Wu Ho-Su Memorial Hospital and Taipei Medical University.
Matsumoto *et al*., 2009 Japan	TG 61.5±7.9 CG 56.4±7.0	Periodontitis: At least 10 sites with PPD≥4 mm Diabetes: HbA1c≥5.5% and FPG≥126 mg/dl	TG (*n=*14) CG (*n=*13)	TG: NSPT + 2% minocycline hydrochloride gel applied into periodontal pockets (every site with PPD ≥4 mm) after NSPT CG: NSPT alone	HbA1c (%) TG 7.1±0.9 CG 7.4±1.1 PPD NA	2 months	HbA1c (%) TG 7.4±0.88 NS CG 7.8±1.27 NS CBG: NS PPD After 2 months, the percentage of sites with PPD≥4 mm in TG reduced by 8.3% (*P≤*0.05) and by 5.0% in CG (*P≤*0.05).	Minocycline improves periodontal disease but has not contributed to a reduction of HbA1c values.	No conflicts of interest. Supported by Grant-in- Aids from Ministry of Education, Culture, Sports, Science and Technology of Japan.
Munenaga *et al*., 2013 Japan	Age range 54-75 years	Periodontitis: High sensitivity c-reactive protein (hsCRP)>500 ng/ml and bone score over 25% constitute mid-to severe- periodontitis. Diabetes: NA	TG (*n=*42) CG (*n=*33)	TG: NSPT + tetracycline– HCl ointment applied into every periodontal pocket (once a week for 4 weeks) combined with sessions of NSPT CG: NSPT alone	HbA1c (%) TG: 7.40±1.17 CG: 7.43±1.17	3 months	HbA1c (%) TG: 6.91±0.86 (*P≤*0.001) CG: 7.13±0.96 (*P≤*0.05) CBG: NA	Treatment using antibiotics is recommended for better metabolic control in subjects with T2D and periodontitis (hsCRP>500 ng/ml).	No conflicts of interest. Supported by 8020 Research fund (2008) from 8020 Promotion Foundation, and Grant-in-Aid by Japan Society for the Promotion of Science.
Priyanka *et al*., 2015 India	TG 40.3±10.2 CG 42.2±8.2	Periodontitis: PPD≥5 mm and/or CAL≥4 mm and vertical bone loss ≥ 3 mm on intraoral periapical radiographs Diabetes: controlled disease with HbA1c<8%	TG (*n=*32) CG (*n=*32)	TG: NSPT + 3% satranidazole gel injected into periodontal pockets after NSPT CG: NSPT + placebo	PPD (mm) TG 8.31±1.42 CG 8.24±1.32	3 months 6 months	PPD (mm) TG 5.32±1.25 (*P≤*0.001) CG 6.55±1.22 (*P≤*0.001) CBG: NA PPD (mm) TG 3.58±1.22 (*P≤*0.001) CG 6.15±1.11 (*P≤*0.001) CBG: NA	Local delivery of 3% Satranidazole into the periodontal pocket stimulated a significant increase in the PPD reduction and CAL gain, compared to placebo gel as an adjunct to NSPT in T2D patients with chronic periodontitis.	No conflicts of interest. Supported by Alkem Laboratories Ltd., Mumbai, India.
Santos *et al*., 2013 Brazil	TG 50.3±9.5 CG 53.9±10.8	Periodontitis: More than 30% of the sites with PPD and CAL≥4 mm Diabetes: History T2D> 5 years	TG (*n=*19) CG (*n=*19)	TG: NSPT + 1% chlorhexidine gel applied into periodontal pockets three times (repeated within 10 minutes) after two appointments of NSPT within 24h CG: NSPT + placebo gel	HbA1c (%) TG 10.0±2.41 CG 10.4±2.9 PPD (mm) TG 3.4±0.5 CG 3.7±0.8	3 months 6 months 12 months	HbA1c (%) TG 9.3±2.75 NS CG 9.7±2.9 NS CBG: NS PPD (mm) TG 2.9±0.4 (*P<*0.05) CG 3.0±0.5 (*P<*0.05) CBG: NS HbA1c (%) TG 9.9±2.40 NS CG 9.6±3.2 NS CBG: NS PPD (mm) TG 2.9±0.4 (*P<*0.05) CG 2.9±0.5 (*P<*0.05) CBG: NS HbA1c (%) TG 9.7±2.54 NS CG 8.9±2.5 NS CBG: NS PPD (mm) TG 3.0±0.4 (*P<*0.05) CG 2.9±0.6 (*P<*0.05) CBG: NS	Treatments did not differ concerning clinical parameters for up to 12 months post-treatment.	No conflicts of interest. Funding: provided by São Paulo State Research Foundation

**Legend:** TG: teste group; CG: Control Group; HbA1c: glycated hemoglobin; NA: not available; FPG: Fasting plasma glucose; PPD: Probing pocket depth; CAL: Clinical attachment level; BoP: Bleeding on probing; T2D: Type 2 Diabetes Mellitus; NSPT: non-surgical periodontal therapy; CBG: Comparison between groups; NS: Non-significant.

**Table 2:** Classification of interventions for HbA1c control at 3 months and performances after 6 months in patients with type 2 diabetes mellitus and periodontitis following the partially contextualized framework for NMA.

**Intervention^1^**	**HbA1c Reduction^2^ MD (95% CI) after 3 Months**	**Certainty of** **Evidence**	**HbA1c Reduction^2^ MD (95% CI) after 6 Months**	**Certainty of Evidence**
Doxy gel	-0.80 (-1.70; 0.10)	Low	0.00 (-1.44; 1.44)	Low
CHX gel	-0.68 (-1.34; -0.02)	Low	-0.53 (-1.57; 0.51)	Very low
Tetra fiber	-0.62 (-0.85; -0.39)	Low	-	-
Tetra ointment	-0.19 (-0.87; 0.49)	Very low	-	-
Minocycline gel	-0.11 (-1.02; 0.80)	Very low	-0.06 (-2.21; 2.09)	Low

**Notes:** Doxy gel: doxycycline gel; CHX gel: chlorhexidine gel; Tetra fiber: tetracycline fiber; Tetra ointment: tetracycline ointment; MD: mean difference; CI: Confidence Interval.

^1^Used as an adjunct of NSPT and compared to NSPT alone.

^2^Results in % HbA1c. MID was considered 0.5%. Negative values mean that the intervention was more effective in reducing HbA1c. Positive values mean that the comparator (NSPT) was more effective.

**Table 3:** Classification of interventions for PPD improvement at 3 months and performances after 6 months in patients with type 2 diabetes mellitus and periodontitis following the partially contextualized framework for NMA.

**Intervention^1^**	**PPD Reduction^2^ MD (95% CI) after 3 Months**	**Certainty of** **Evidence**	**PPD Reduction^2^ MD (95% CI) after 6 Months**	**Certainty of Evidence**
STZ gel	-1.30 (-2.22; -0.38)	Low	-2.64 (-3.56; -1.72)	Moderate
CLM gel	-1.01 (-1.50; -0.52)	Low	-0.98 (-1.51; -0.45)	Low
Tetra fiber	-0.92 (-1.66; -0.18)	Very low	-	-
AZT gel	-0.26 (-0.87; 0.35)	Low	-0.23 (-0.77; 0.31)	Low
Minocycline gel	0.05 (-0.81; 0.91)	Very low	0.00 (-0.87; 0.87)	Very low
Doxy gel	0.00 (-0.54; 0.54)	Moderate	0.10 (-0.47; 0.67)	Moderate
CHX gel	0.00 (-0.38; 0.38)	Very low	0.09 (-0.30; 0.48)	Very low

**Notes:** STZ gel: satranidazole gel; CLM gel: clarithromycin gel; Tetra fiber: tetracycline fiber; AZT gel: azithromycin gel; Doxy gel: doxycycline gel; CHX gel: chlorhexidine gel; MD: mean difference; CI: Confidence Interval.

^1^Used as an adjunct of NSPT and compared to NSPT alone.

^2^Results in mm. MID was considered ≥ 1mm. Negative values mean that the intervention was more effective in reducing PPD. Positive values mean that the comparator [NSPT] was more effective.

## Data Availability

All data generated or analysed during this study are included in this published article and its supplementary materials.

## LIST OF ABBREVIATIONS

MD: Mean Differences
NMA: Network Meta-Analysis
NSPT: Non-Surgical Periodontal Therapy
RCT: Randomized Clinical Trials

## STANDARDS OF REPORTING

PRISMA guidelines and methodology were followed.

## SUPPLEMENTARY MATERIAL

Supplementary File **S1**: Complete search strategy for each database. Supplementary File **S2** - Excluded articles and reasons for exclusion. Supplementary File **S3** - League table for each comparison (Mean Differences). Supplementary File **S4** - Assessment of inconsistency for all studies (MD). Supplementary File **S5** - GRADE explanations and results (containing Supplementary Tables S1, S2, S3 and S4). Supplementary File **S6** - PRISMA NMA Checklist.

## References

[1] Nguyen A.T.M., Akhter R., Garde S. (2020). The association of periodontal disease with the complications of diabetes mellitus. A systematic review.. Diabetes Res. Clin. Pract..

[2] Nascimento G.G., Leite F.R.M., Vestergaard P., Scheutz F., López R. (2018). Does diabetes increase the risk of periodontitis? A systematic review and meta-regression analysis of longitudinal prospective studies.. Acta Diabetol..

[3] Baeza M., Morales A., Cisterna C. (2020). Effect of periodontal treatment in patients with periodontitis and diabetes: Systematic review and meta-analysis.. J. Appl. Oral Sci..

[4] Casanova L., Hughes F.J., Preshaw P.M. (2014). Diabetes and periodontal disease: A two-way relationship.. Br. Dent. J..

[5] Llambés F., Arias-Herrera S., Caffesse R. (2015). Relationship between diabetes and periodontal infection.. World J. Diabetes.

[6] Preshaw P.M., Alba A.L., Herrera D. (2012). Periodontitis and diabetes: A two-way relationship.. Diabetologia.

[7] Teeuw W.J., Gerdes V.E.A., Loos B.G. (2010). Effect of periodontal treatment on glycemic control of diabetic patients: A systematic review and meta-analysis.. Diabetes Care.

[8] Herrera D., Matesanz P., Bascones-Martínez A., Sanz M. (2012). Local and systemic antimicrobial therapy in periodontics.. J. Evid. Based Dent. Pract..

[9] Matesanz-Pérez P., García-Gargallo M., Figuero E., Bascones-Martínez A., Sanz M., Herrera D. (2013). A systematic review on the effects of local antimicrobials as adjuncts to subgingival debridement, compared with subgingival debridement alone, in the treatment of chronic periodontitis.. J. Clin. Periodontol..

[10] Cobb C.M., Sottosanti J.S. (2021). A re‐evaluation of scaling and root planing.. J. Periodontol..

[11] Mombelli A., Samaranayake L.P. (2004). Topical and systemic antibiotics in the management of periodontal diseases.. Int. Dent. J..

[12] Ramanauskaite E., Machiulskiene V. (2020). Antiseptics as adjuncts to scaling and root planing in the treatment of periodontitis: A systematic literature review.. BMC Oral Health.

[13] Herrera D., Matesanz P., Martín C., Oud V., Feres M., Teughels W. (2020). Adjunctive effect of locally delivered antimicrobials in periodontitis therapy: A systematic review and meta‐analysis.. J. Clin. Periodontol..

[14] Rovai E.S., Souto M.L.S., Ganhito J.A., Holzhausen M., Chambrone L., Pannuti C.M. (2016). Efficacy of local antimicrobials in the non‐surgical treatment of patients with periodontitis and diabetes: A systematic review.. J. Periodontol..

[15] Santos C.M.M.L., Lira-Junior R., Fischer R.G., Santos A.P.P., Oliveira B.H. (2015). Systemic antibiotics in periodontal treatment of diabetic patients: A systematic review.. PLoS One.

[16] O’Connell P.A.A., Taba M., Nomizo A. (2008). Effects of periodontal therapy on glycemic control and inflammatory markers.. J. Periodontol..

[17] Altamash M., Klinge B., Engström P.E. (2016). Periodontal treatment and H b A 1c levels in subjects with diabetes mellitus.. J. Oral Rehabil..

[18] Grossi S.G., Skrepcinski F.B., DeCaro T., Zambon J.J., Cummins D., Genco R.J. (1996). Response to periodontal therapy in diabetes and smokers.. J. Periodontol..

[19] Oswal S.K., Dwarkanath C.D., Ramesh A.V. (2011). Evaluation of periodontal surgical procedures in type-2 diabetic patients.. Int. J. Stomatol. Occlusion Med..

[20] Haas A.N., Furlaneto F., Gaio E.J. (2021). New tendencies in non-surgical periodontal therapy.. Braz. Oral Res..

[21] Hutton B., Salanti G., Caldwell D.M. (2015). The PRISMA extension statement for reporting of systematic reviews incorporating network meta-analyses of health care interventions: checklist and explanations.. Ann. Intern. Med..

[22] Tonetti M.S., Greenwell H., Kornman K.S. (2018). Staging and grading of periodontitis: Framework and proposal of a new classification and case definition.. J. Periodontol..

[23] Committee I.E. (2009). International expert committee report on the role of the A1C assay in the diagnosis of diabetes.. Diabetes Care.

[24] Ouzzani M., Hammady H., Fedorowicz Z., Elmagarmid A. (2016). Rayyan—a web and mobile app for systematic reviews.. Syst. Rev..

[25] Higgins J.P., Savović J., Page M.J., Elbers R.G., Sterne J.A. (2021). Assessing risk of bias in a randomized trial, Cochrane Training..

[26] Lamont T.J., Clarkson J.E., Ricketts D.N.J., Heasman P.A., Ramsay C.R., Gillies K. (2021). Developing a core outcome set for periodontal trials.. PLoS One.

[27] Owen R.K., Bradbury N., Xin Y., Cooper N., Sutton A. (2019). MetaInsight: An interactive web‐based tool for analyzing, interrogating, and visualizing network meta‐analyses using R‐shiny and netmeta.. Res. Synth. Methods.

[28] Puhan MA, Schünemann HJ, Murad MH (2014). A GRADE working group approach for rating the quality of treatment effect estimates from network meta-analysis.. BMJ.

[29] Brignardello-Petersen R., Bonner A., Alexander P.E. (2018). Advances in the GRADE approach to rate the certainty in estimates from a network meta-analysis.. J. Clin. Epidemiol..

[30] Araújo E.G., Oliveira D.M.S.L., Martins C.C., Stefani C.M. (2022). Efficacy of antioxidant supplementation to non-surgical periodontal therapy on metabolic control in type 2 diabetes patients: A network meta-analysis.. Antioxidants.

[31] Martins C.C., Firmino R.T., Riva J.J. (2020). Desensitizing toothpastes for dentin hypersensitivity: A network meta-analysis.. J. Dent. Res..

[32] Brignardello-Petersen R., Izcovich A., Rochwerg B. (2020). GRADE approach to drawing conclusions from a network meta-analysis using a partially contextualised framework.. BMJ.

[33] Carrasco-Labra A., Devji T., Qasim A. (2021). Minimal important difference estimates for patient-reported outcomes: A systematic survey.. J. Clin. Epidemiol..

[34] Agarwal E., Bajaj P., Naik S.B., Pradeep A.R. (2017). Locally delivered 0.5% azithromycin as an adjunct to non‐surgical treatment in patients with chronic periodontitis with type 2 diabetes: A randomized controlled clinical trial.. J. Periodontol..

[35] Bajaj P., Pradeep A.R., Agarwal E., Kumari M., Naik S.B. (2012). Locally delivered 0.5% clarithromycin, as an adjunct to nonsurgical treatment in chronic periodontitis with well‐controlled type 2 diabetes: A randomized controlled clinical trial.. J. Investig. Clin. Dent..

[36] Dodwad V., Ahuja S., Kukreja B.J. (2012). Effect of locally delivered tetracycline hydrochloride as an adjunct to scaling and root planing on Hba1c, C-reactive protein, and lipid profile in type 2 diabetes: A clinico-biochemical study.. Contemp. Clin. Dent..

[37] Priyanka N., Kalra N., Saquib S. (2015). Efficacy of subgingivally delivered satranidazole in the treatment of type 2 diabetes subjects with chronic periodontitis: A randomized controlled clinical trial.. J. Int. Acad. Periodontol..

[38] Faramarzi M., Shirmohammadi A., Chitsazi M., Shamami M.S., Ghanitab S. (2017). The clinical and metabolic effects of subgingival application of xanthan-based chlorhexidine gel in Type 2 diabetic patients with chronic periodontitis.. Dent. Res. J..

[39] Lecio G., Ribeiro F.V., Pimentel S.P. (2020). Novel 20% doxycycline-loaded PLGA nanospheres as adjunctive therapy in chronic periodontitis in type-2 diabetics: randomized clinical, immune and microbiological trial.. Clin. Oral Investig..

[40] Santos V.R., Lima J.A., Miranda T.S. (2013). Full‐mouth disinfection as a therapeutic protocol for type‐2 diabetic subjects with chronic periodontitis: Twelve‐month clinical outcomes. A randomized controlled clinical trial.. J. Clin. Periodontol..

[41] Lin S.J., Tu Y.K., Tsai S.C., Lai S.M., Lu H.K. (2012). Non-surgical periodontal therapy with and without subgingival minocycline administration in patients with poorly controlled type II diabetes: A randomized controlled clinical trial.. Clin. Oral Investig..

[42] Matsumoto S., Ogawa H., Soda S., Hirayama S., Amarasena N., Aizawa Y. (2009). Effect of antimicrobial periodontal treatment and maintenance on serum adiponectin in type 2 diabetes mellitus.. J. Clin. Periodontol..

[43] Munenaga Y., Yamashina T., Tanaka J., Nishimura F. (2013). Improvement of glycated hemoglobin in Japanese subjects with type 2 diabetes by resolution of periodontal inflammation using adjunct topical antibiotics: Results from the Hiroshima Study.. Diabetes Res. Clin. Pract..

[44] Parihar S., Kesarwani S., Singh S., Gautam A., Pandey A., Anjum M. (2022). A new era of Nano!!! Comparative evaluation of ganglioside polymeric nanoparticle coated satranidazole gel and 1% metronidazole gel for the treatment of periodontitis.. J. Indian Soc. Periodontol..

[45] Priyanka N., Kalra N., Saquib S. (2015). Clinical and microbiological efficacy of 3% satranidazole gel as a local drug delivery system in the treatment of chronic periodontitis: A randomized, controlled clinical trial.. Contemp. Clin. Dent..

[46] Bansal K., Rawat M.K., Jain A., Rajput A., Chaturvedi T.P., Singh S. (2009). Development of satranidazole mucoadhesive gel for the treatment of periodontitis.. AAPS PharmSciTech.

[47] Tilakaratne A., Soory M. (2014). Anti-inflammatory actions of adjunctive tetracyclines and other agents in periodontitis and associated comorbidities.. Open Dent. J..

[48] Sharma D., Das A.C., Das S.J., Panda S., Taschieri S., Fabbro M.D. (2019). Adjunctive effect of doxycycline with conventional periodontal therapy on glycemic level for chronic periodontitis with type 2 diabetes mellitus subjects.. J. Contemp. Dent. Pract..

[49] Rao S.K., Setty S., Acharya A.B., Thakur S.L. (2012). Efficacy of locally‐delivered doxycycline microspheres in chronic localized periodontitis and on Porphyromonas gingivalis.. J. Investig. Clin. Dent..

[50] Vandana K.L., Javali M.A. (2012). A comparative evaluation of atrigel delivery system (10% doxycycline hyclate) Atridox with scaling and root planing and combination therapy in treatment of periodontitis: A clinical study.. J. Indian Soc. Periodontol..

[51] Jalaluddin M., Ahamed S., Khalid I., Moon N., Shafi T.K., Ali F.M. (2013). The use of controlled release locally delivered 10% doxycycline hyclate gel as an adjunct to scaling and root planing in the treatment of chronic periodontitis: Clinical and microbiological results.. J. Contemp. Dent. Pract..

[52] Shelke A., Gadhiya N., Narkhede S., Laddha R., Mahajania M., Shetty G.P. (2018). Effect of subgingival doxycycline placement on clinical and microbiological parameters in inflammatory periodontal disease: Both in vivo and in vitro studies.. J. Contemp. Dent. Pract..

[53] Martorelli de Lima A.F., Cury C.C., Palioto D.B., Duro A.M., Silva R.C., Wolff L.F. (2004). Therapy with adjunctive doxycycline local delivery in patients with type 1 diabetes mellitus and periodontitis.. J. Clin. Periodontol..

[54] Nath S., Pulikkotil S., Dharmarajan L., Arunachalam M., Jing K. (2020). Effect of locally delivered doxycycline as an adjunct to scaling and root planing in the treatment of periodontitis in smokers: A systematic review of randomized controlled trials with meta-analysis and trial sequential analysis.. Dent. Res. J..

[55] Al-Ahmari M.M. (2022). Effect of antibiotics and carbohydrate diet control instructions in improving glycated hemoglobin among type ii diabetic patients with periodontitis.. J. Contemp. Dent. Pract..

[56] Al Mubarak S., Abou Rass M., Alsuwyed A. (2010). A new paradigm between mechanical scaling and root planing combined with adjunctive chemotherapy for glycated hemoglobin improvement in diabetics.. Int. J. Diabetes Mellit..

[57] Gomaa A., El G., Mahmoud A., El-Zamrany A. (2018). Adjunctive subantimicrobial dose doxycycline in the treatment of chronic periodontitis in type 2 diabetic patients: A unique combination therapy.. Balk. J. Dent. Med..

[58] Paolantonio M., D’Ercole S., Pilloni A. (2009). Clinical, microbiologic, and biochemical effects of subgingival administration of a Xanthan-based chlorhexidine gel in the treatment of periodontitis: A randomized multicenter trial.. J. Periodontol..

[59] Srirangarajan S., Setty R., Satyanarayan A., Shetty S. (2016). Effect of full-mouth disinfection on insulin sensitivity in type 2 diabetes patients with and without chronic periodontitis.. Quintessence Int..

[60] Zhao H., Hu J., Zhao L. (2020). Adjunctive subgingival application of Chlorhexidine gel in nonsurgical periodontal treatment for chronic periodontitis: A systematic review and meta-analysis.. BMC Oral Health.

[61] Panwar M., Gupta S.H. (2009). Local drug delivery with tetracycline fiber: An alternative to surgical periodontal therapy.. Med. J. Armed Forces India.

[62] Goodson J.M., Holborow D., Dunn R.L., Hogan P., Dunham S. (1983). Monolithic tetracycline-containing fibers for controlled delivery to periodontal pockets.. J. Periodontol..

[63] Aimetti M., Romano F., Torta I., Cirillo D., Caposio P., Romagnoli R. (2004). Debridement and local application of tetracycline‐loaded fibres in the management of persistent periodontitis: results after 12 months.. J. Clin. Periodontol..

[64] Sinha S., Kumar S., Dagli N., Dagli R. (2014). Effect of tetracycline HCl in the treatment of chronic periodontitis - A clinical study.. J. Int. Soc. Prev. Community Dent..

[65] Kafle S., Pradhan S., Gupta S. (2018). Locally delivered tetracycline fibres in the treatment of chronic periodontitis.. JNSSPOI.

[66] Christersson L.A., Norderyd O.M., Puchalsky C.S. (1993). Topical application of tetracycline-HCI in human periodontitis Christersson LA, Norderyd OM and Puchalsky CS: Topical application of tetra-cycUne-HCl in human periodontitis.. J. Clin. Periodontol..

[67] Pavia M., Nobile C.G.A., Angelillo I.F. (2003). Meta-analysis of local tetracycline in treating chronic periodontitis.. J. Periodontol..

[68] Gyssens I.C. (2001). Quality measures of antimicrobial drug use.. Int. J. Antimicrob. Agents.

[69] Marconcini S., Giammarinaro E., Cosola S., Oldoini G., Genovesi A., Covani U. (2021). Effects of non-surgical periodontal treatment on reactive oxygen metabolites and glycemic control in diabetic patients with chronic periodontitis.. Antioxidants.

[70] Corbella S., Francetti L., Taschieri S., De Siena F., Fabbro M.D. (2013). Effect of periodontal treatment on glycemic control of patients with diabetes: A systematic review and meta‐analysis.. J. Diabetes Investig..

[71] Simpson T.C., Clarkson J.E., Worthington H.V. (2022). Treatment of periodontitis for glycaemic control in people with diabetes mellitus.. Cochrane Database Syst. Rev..

[72] Garcia D., Tarima S., Okunseri C. (2015). Periodontitis and glycemic control in diabetes: NHANES 2009 to 2012.. J. Periodontol..

[73] Guyatt G.H., Oxman A.D., Vist G.E. (2008). GRADE: An emerging consensus on rating quality of evidence and strength of recommendations.. BMJ.

