# Supplementary material for: Efficacy of Adjunctive Local Antimicrobials to Non-Surgical Periodontal Therapy in Pocket Reduction and Glycemic Control of Patients with Type 2 Diabetes: A Network Meta-Analysis
Source: Curr Diabetes Rev. 2024 Sep 3;21(7):E15733998320667. doi: 10.2174/0115733998320667240805045742 (PMC12082567; doi:10.2174/0115733998320667240805045742)
Supplement: Supplementary file 1 [file CDR-21-7-E15733998320667_SD1.zip › CDR-21-7-E15733998320667_SD1/S1 - Complete search strategy for each database.docx]

**Supplementary file 1 – Complete search strategy for each database**

| **Database** | **Search**  1^st^ search: 2022-02-04  2^nd^ search: 2024-02-25 | **References** |
| --- | --- | --- |
| **PubMed** | ("Diabetes Mellitus"[All Fields] OR "Type 2 Diabetes Mellitus"[All Fields] OR "Diabetes"[All Fields] OR "Metabolic Diseases"[All Fields] OR "Hyperglycemia"[All Fields] OR "Glucose Metabolism Disorders"[All Fields] OR "Metabolic Control"[All Fields] OR "Glycated Hemoglobin A"[All Fields] OR "Glycated Hemoglobin"[All Fields] OR "HbA1"[All Fields] OR "Glycosylated Hemoglobin A"[All Fields] OR "Blood Glucose"[All Fields] OR "Glycemic Index"[All Fields] OR "Fasting blood glucose"[All Fields] OR "Post-prandial blood glucose"[All Fields] OR "diabetes mellitus type 2"[All Fields] OR "diabetes type 2"[All Fields] OR "type II diabetes mellitus"[All Fields] OR "type 2 diabetes"[All Fields] OR "type II diabetes"[All Fields] OR "advanced glycation end products"[All Fields] OR "HbA1c"[All Fields] OR (("human s"[All Fields] OR "humans"[MeSH Terms] OR "humans"[All Fields] OR "human"[All Fields]) AND ("Glycated Hemoglobin A"[MeSH Terms] OR "Glycated Hemoglobin A"[All Fields] OR ("hemoglobin"[All Fields] AND "a1c"[All Fields]) OR "hemoglobin a1c"[All Fields]) AND ("protein s"[All Fields] OR "proteinous"[All Fields] OR "proteins"[MeSH Terms] OR "proteins"[All Fields] OR "protein"[All Fields])) OR "glycohemoglobin A"[All Fields] OR (("glycosyl"[All Fields] OR "glycosylate"[All Fields] OR "glycosylated"[All Fields] OR "glycosylates"[All Fields] OR "glycosylating"[All Fields] OR "glycosylation"[MeSH Terms] OR "glycosylation"[All Fields] OR "glycosylations"[All Fields] OR "glycosylic"[All Fields] OR "glycosyls"[All Fields]) AND ("analysis"[MeSH Subheading] OR "analysis"[All Fields]) AND ("haemoglobin a"[All Fields] OR "hemoglobin a"[MeSH Terms] OR "hemoglobin a"[All Fields])) OR "glycated hemoglobins"[All Fields] OR "Glycated Hemoglobin"[All Fields] OR "blood glucose metabolism"[All Fields] OR "blood glucose analysis"[All Fields]) AND (("local"[All Fields] OR "subgingival"[All Fields] OR "subgingivally delivered"[All Fields] OR "subgingival delivery"[All Fields] OR "subgingival application"[All Fields] OR "locally delivered"[All Fields] OR "local use"[All Fields] OR "gel"[All Fields] OR "topical"[All Fields] OR "delivery vehicle"[All Fields] OR "nanospheres"[All Fields]) AND ("antibiotic"[All Fields] OR "antibiotics"[All Fields] OR "antimicrobial"[All Fields] OR "antimicrobials"[All Fields] OR "anti-bacterial"[All Fields] OR "agents"[All Fields] OR "anti-bacterial agents"[All Fields] OR "antibacterial agents"[All Fields] OR "disinfection"[All Fields] OR "amoxicillin"[All Fields] OR "amox"[All Fields] OR "amoxicilline"[All Fields] OR "penicillin"[All Fields] OR "Azithromycin"[All Fields] OR "clarithromycin"[All Fields] OR "doxycycline"[All Fields] OR "doxycyclin"[All Fields] OR "Tetracycline"[All Fields] OR "minocycline"[All Fields] OR "clindamycin"[All Fields] OR "Fluoroquinolones"[All Fields] OR "metronidazole"[All Fields] OR "Chlorhexidine"[All Fields] OR "triclosan"[All Fields] OR "Cetylpyridinium"[All Fields] OR "Cetylpyridinium chloride"[All Fields] OR "CPC"[All Fields] OR "povidone iodine"[All Fields])) AND ("Chronic Periodontitis"[All Fields] OR ("Chronic Periodontitis"[MeSH Terms] OR ("chronic"[All Fields] AND "Periodontitis"[All Fields]) OR "Chronic Periodontitis"[All Fields] OR ("chronic"[All Fields] AND "Periodontitides"[All Fields])) OR "Adult Periodontitis"[All Fields] OR ("Chronic Periodontitis"[MeSH Terms] OR ("chronic"[All Fields] AND "Periodontitis"[All Fields]) OR "Chronic Periodontitis"[All Fields] OR ("adult"[All Fields] AND "Periodontitides"[All Fields])) OR "Periodontal treatment"[All Fields] OR "Periodontal therapy"[All Fields] OR "nonsurgical periodontal therapy"[All Fields] OR "non-surgical periodontal therapy"[All Fields] OR "Periodontitis"[All Fields] OR "Periodontitides"[All Fields] OR "Periodontal Diseases"[All Fields] OR "Periodontal Disease"[All Fields] OR "Parodontosis"[All Fields] OR "Parodontoses"[All Fields] OR "Pyorrhea Alveolaris"[All Fields] OR "scaling and root planing"[All Fields] OR "Periodontal Pocket"[All Fields] OR "periodontal pockets"[All Fields] OR "pocket depth"[All Fields] OR "pocket probing"[All Fields] OR "probing depth"[All Fields] OR "periodontal health"[All Fields] OR "Alveolar Bone Loss"[All Fields] OR "Alveolar Bone Losses"[All Fields] OR "Alveolar Resorption"[All Fields] OR "Periodontal Bone Losses"[All Fields] OR "Periodontal Bone Loss"[All Fields] OR "Periodontal Resorption"[All Fields] OR (("clinical trials, phase i as topic"[MeSH Terms] OR "phase i as topic clinical trials"[All Fields] OR "phase 1"[All Fields]) AND ("periodontal"[All Fields] OR "periodontally"[All Fields] OR "periodontically"[All Fields] OR "periodontics"[MeSH Terms] OR "periodontics"[All Fields] OR "periodontic"[All Fields] OR "Periodontitis"[MeSH Terms] OR "Periodontitis"[All Fields] OR "Periodontitides"[All Fields]) AND ("therapeutics"[MeSH Terms] OR "therapeutics"[All Fields] OR "therapies"[All Fields] OR "therapy"[MeSH Subheading] OR "therapy"[All Fields] OR "therapy s"[All Fields] OR "therapys"[All Fields])) OR "initial periodontal therapy"[All Fields] OR "Periodontal Index"[All Fields] OR "Periodontal Indices"[All Fields] OR "Periodontal Indexes"[All Fields] OR "bleeding on probing"[All Fields] OR "bleeding index"[All Fields] OR "periodontal inflammation"[All Fields] OR "Gingival Bleeding on Probing"[All Fields] OR "gingival bleeding"[All Fields] OR "Gingival Index"[All Fields] OR "Gingival Indices"[All Fields] OR "Gingival Indexes"[All Fields] OR "Dental Plaque Index"[All Fields] OR "Dental Plaque Indexes"[All Fields] OR "Dental Plaque Indices"[All Fields] OR "Dental Plaque"[All Fields] OR "dental biofilm"[All Fields] OR "dental biofilms"[All Fields] OR "Periodontal probing depth"[All Fields] OR "Clinical attachment loss"[All Fields] OR "Periodontal Debridement"[All Fields] OR "Periodontal Debridements"[All Fields] OR "Periodontal Pocket Debridement"[All Fields] OR "Periodontal therapy"[All Fields] OR "Dental Scaling"[All Fields] OR "Root Scaling"[All Fields] OR "Subgingival Scaling"[All Fields] OR "Periodontal treatment"[All Fields] OR "Periodontal therapy"[All Fields] OR "nonsurgical periodontal treatment"[All Fields] OR "non-surgical periodontal treatment"[All Fields] OR "non-surgical mechanical therapy"[All Fields] OR "Root Planing"[All Fields] OR "Root Planings"[All Fields] OR "periodontal attachment loss"[All Fields] OR "clinical attachment level"[All Fields] OR "attachment gain"[All Fields] OR "periodontal healing"[All Fields] OR "periodontal diseased sites"[All Fields] OR "periodontal parameters"[All Fields] OR "periodontal clinical parameters"[All Fields] OR "pocket closure"[All Fields] OR "pocket depth reduction"[All Fields] OR "residual pockets"[All Fields] OR "periodontal recession"[All Fields] OR "Gingival Recession"[All Fields] OR "Gingival Recessions"[All Fields] OR (("periodontal"[All Fields] OR "periodontally"[All Fields] OR "periodontically"[All Fields] OR "periodontics"[MeSH Terms] OR "periodontics"[All Fields] OR "periodontic"[All Fields] OR "Periodontitis"[MeSH Terms] OR "Periodontitis"[All Fields] OR "Periodontitides"[All Fields]) AND "re instrumentation"[All Fields]) OR (("pocket"[All Fields] OR "pocket s"[All Fields] OR "pocketing"[All Fields] OR "pockets"[All Fields]) AND "re instrumentation"[All Fields]) OR (("periodontal"[All Fields] OR "periodontally"[All Fields] OR "periodontically"[All Fields] OR "periodontics"[MeSH Terms] OR "periodontics"[All Fields] OR "periodontic"[All Fields] OR "Periodontitis"[MeSH Terms] OR "Periodontitis"[All Fields] OR "Periodontitides"[All Fields]) AND ("residual"[All Fields] OR "residuals"[All Fields]) AND ("site s"[All Fields] OR "sited"[All Fields] OR "sites"[All Fields] OR "siting"[All Fields] OR "sitings"[All Fields]))) | 1^st^ search 130  2^nd^ search  157 |
| **Cochrane** | **(("Diabetes Mellitus" OR "Type 2 Diabetes Mellitus" OR "Diabetes" OR "Metabolic Diseases" OR "Hyperglycemia" OR "Glucose Metabolism Disorders" OR "Metabolic Control" OR "Glycated Hemoglobin A" OR "Glycated Hemoglobin" OR "HbA1" OR "Glycosylated Hemoglobin A" OR "Blood Glucose" OR "Glycemic Index" OR "Fasting blood glucose" OR "Post-prandial blood glucose" OR "diabetes mellitus type 2" OR "diabetes type 2" OR "type II diabetes mellitus" OR "type 2 diabetes" OR "type II diabetes" OR "advanced glycation end products" OR "HbA1c" OR "human hemoglobin A1c protein" OR "glycohemoglobin A" OR "glycosylated analysis hemoglobin A" OR "glycated hemoglobins" OR "glycated hemoglobin" OR "blood glucose metabolism" OR "blood glucose analysis")):ti,ab,kw AND (("local" OR "subgingival" OR "subgingivally delivered" OR "subgingival delivery" OR "subgingival application" OR "locally delivered" OR "local use" OR "gel" OR "topical" OR "delivery vehicle" OR "nanospheres") AND ("antibiotic" OR "antibiotics" OR "antimicrobial" OR "antimicrobials" OR "anti-bacterial" OR "agents" OR "anti-bacterial agents" OR "antibacterial agents" OR "disinfection" OR "amoxicillin" OR "amox" OR "amoxicilline" OR "penicillin" OR "Azithromycin" OR "clarithromycin" OR "doxycycline" OR "doxycyclin" OR "Tetracycline" OR "minocycline" OR "clindamycin" OR "Fluoroquinolones" OR "metronidazole" OR "Chlorhexidine" OR "triclosan" OR "Cetylpyridinium" OR "Cetylpyridinium chloride" OR "CPC" OR "povidone iodine")):ti,ab,kw AND (("Chronic Periodontitis" OR "Chronic Periodontitides" OR "Adult Periodontitis" OR "Adult Periodontitides" OR "Periodontal treatment" OR "Periodontal therapy" OR "nonsurgical periodontal therapy" OR "non-surgical periodontal therapy" OR "Periodontitis" OR "Periodontitides" OR "Periodontal Diseases" OR "Periodontal Disease" OR "Parodontosis" OR "Parodontoses" OR "Pyorrhea Alveolaris" OR "scaling and root planing" OR "Periodontal Pocket" OR "periodontal pockets" OR "pocket depth" OR "pocket probing" OR "probing depth" OR "periodontal health" OR "Alveolar Bone Loss" OR "Alveolar Bone Losses" OR "Alveolar Resorption" OR "Periodontal Bone Losses" OR "Periodontal Bone Loss" OR "Periodontal Resorption" OR "phase 1 periodontal therapy" OR "initial periodontal therapy" OR "Periodontal Index" OR "Periodontal Indices" OR "Periodontal Indexes" OR "bleeding on probing" OR "bleeding index" OR "periodontal inflammation" OR "Gingival Bleeding on Probing" OR "gingival bleeding" OR "Gingival Index" OR "Gingival Indices" OR "Gingival Indexes" OR "Dental Plaque Index" OR "Dental Plaque Indexes" OR "Dental Plaque Indices" OR "Dental Plaque" OR "dental biofilm" OR "dental biofilms" OR "Periodontal probing depth" OR "Clinical attachment loss" OR "Periodontal Debridement" OR "Periodontal Debridements" OR "Periodontal Pocket Debridement" OR "periodontal therapy" OR "Dental Scaling" OR "Root Scaling" OR "Subgingival Scaling" OR "periodontal treatment" OR "periodontal therapy" OR "nonsurgical periodontal treatment" OR "non-surgical periodontal treatment" OR "non-surgical mechanical therapy" OR "Root Planing" OR "Root Planings" OR "periodontal attachment loss" OR "clinical attachment level" OR "attachment gain" OR "periodontal healing" OR "periodontal diseased sites" OR "periodontal parameters" OR "periodontal clinical parameters" OR "pocket closure" OR "pocket depth reduction" OR "residual pockets" OR "periodontal recession" OR "Gingival Recession" OR "Gingival Recessions" OR "periodontal re-instrumentation" OR "pocket re-instrumentation" OR "periodontal residual sites")):ti,ab,kw** | 1^st^ search 129  2^nd^ search  168 |
| **Embase** | (‘Diabetes Mellitus’ OR ‘Type 2 Diabetes Mellitus’ OR ‘Diabetes’ OR ‘Metabolic Diseases’ OR ‘Hyperglycemia’ OR ‘Glucose Metabolism Disorders’ OR ‘Metabolic Control’ OR ‘Glycated Hemoglobin A’ OR ‘Glycated Hemoglobin’ OR ‘HbA1’ OR ‘Glycosylated Hemoglobin A’ OR ‘Blood Glucose’ OR ‘Glycemic Index’ OR ‘Fasting blood glucose’ OR ‘Post-prandial blood glucose’ OR ‘diabetes mellitus type 2’ OR ‘diabetes type 2’ OR ‘type II diabetes mellitus’ OR ‘type 2 diabetes’ OR ‘type II diabetes’ OR ‘advanced glycation end products’ OR ‘HbA1c’ OR ‘human hemoglobin A1c protein’ OR ‘glycohemoglobin A’ OR ‘glycosylated analysis hemoglobin A’ OR ‘glycated hemoglobins’ OR ‘glycated hemoglobin’ OR ‘blood glucose metabolism’ OR ‘blood glucose analysis’) AND (‘local’ OR ‘subgingival’ OR ‘subgingivally delivered’ OR ‘subgingival delivery’ OR ‘subgingival application’ OR ‘locally delivered’ OR ‘local use’ OR ‘gel’ OR ‘topical’ OR ‘delivery vehicle’ OR ‘nanospheres’) AND (‘antibiotic’ OR ‘antibiotics’ OR ‘antimicrobial’ OR ‘antimicrobials’ OR ‘anti-bacterial’ OR ‘agents’ OR ‘anti-bacterial agents’ OR ‘antibacterial agents’ OR ‘disinfection’ OR ‘amoxicillin’ OR ‘amox’ OR ‘amoxicilline’ OR ‘penicillin’ OR ‘Azithromycin’ OR ‘clarithromycin’ OR ‘doxycycline’ OR ‘doxycyclin’ OR ‘Tetracycline’ OR ‘minocycline’ OR ‘clindamycin’ OR ‘Fluoroquinolones’ OR ‘metronidazole’ OR ‘Chlorhexidine’ OR ‘triclosan’ OR ‘Cetylpyridinium’ OR ‘Cetylpyridinium chloride’ OR ‘CPC’ OR ‘povidone iodine’) AND (‘Chronic Periodontitis’ OR ‘Chronic Periodontitides’ OR ‘Adult Periodontitis’ OR ‘Adult Periodontitides’ OR ‘Periodontal treatment’ OR ‘Periodontal therapy’ OR ‘nonsurgical periodontal therapy’ OR ‘non-surgical periodontal therapy’ OR ‘Periodontitis’ OR ‘Periodontitides’ OR ‘Periodontal Diseases’ OR ‘Periodontal Disease’ OR ‘Parodontosis’ OR ‘Parodontoses’ OR ‘Pyorrhea Alveolaris’ OR ‘scaling and root planing’ OR ‘Periodontal Pocket’ OR ‘periodontal pockets’ OR ‘pocket depth’ OR ‘pocket probing’ OR ‘probing depth’ OR ‘periodontal health’ OR ‘Alveolar Bone Loss’ OR ‘Alveolar Bone Losses’ OR ‘Alveolar Resorption’ OR ‘Periodontal Bone Losses’ OR ‘Periodontal Bone Loss’ OR ‘Periodontal Resorption’ OR ‘phase 1 periodontal therapy’ OR ‘initial periodontal therapy’ OR ‘Periodontal Index’ OR ‘Periodontal Indices’ OR ‘Periodontal Indexes’ OR ‘bleeding on probing’ OR ‘bleeding index’ OR ‘periodontal inflammation’ OR ‘Gingival Bleeding on Probing’ OR ‘gingival bleeding’ OR ‘Gingival Index’ OR ‘Gingival Indices’ OR ‘Gingival Indexes’ OR ‘Dental Plaque Index’ OR ‘Dental Plaque Indexes’ OR ‘Dental Plaque Indices’ OR ‘Dental Plaque’ OR ‘dental biofilm’ OR ‘dental biofilms’ OR ‘Periodontal probing depth’ OR ‘Clinical attachment loss’ OR ‘Periodontal Debridement’ OR ‘Periodontal Debridements’ OR ‘Periodontal Pocket Debridement’ OR ‘periodontal therapy’ OR ‘Dental Scaling’ OR ‘Root Scaling’ OR ‘Subgingival Scaling’ OR ‘periodontal treatment’ OR ‘periodontal therapy’ OR ‘nonsurgical periodontal treatment’ OR ‘non-surgical periodontal treatment’ OR ‘non-surgical mechanical therapy’ OR ‘Root Planing’ OR ‘Root Planings’ OR ‘periodontal attachment loss’ OR ‘clinical attachment level’ OR ‘attachment gain’ OR ‘periodontal healing’ OR ‘periodontal diseased sites’ OR ‘periodontal parameters’ OR ‘periodontal clinical parameters’ OR ‘pocket closure’ OR ‘pocket depth reduction’ OR ‘residual pockets’ OR ‘periodontal recession’ OR ‘Gingival Recession’ OR ‘Gingival Recessions’ OR ‘periodontal re-instrumentation’ OR ‘pocket re-instrumentation’ OR ‘periodontal residual sites’) | 1^st^ search 812  2^nd^ search  989 |
| **LIVIVO** | ("Diabetes Mellitus" OR "Type 2 Diabetes Mellitus" OR "Diabetes" OR "Metabolic Diseases" OR "Hyperglycemia" OR "Glucose Metabolism Disorders" OR "Metabolic Control" OR "Glycated Hemoglobin A" OR "Glycated Hemoglobin" OR "HbA1" OR "Glycosylated Hemoglobin A" OR "Blood Glucose" OR "Glycemic Index" OR "Fasting blood glucose" OR "Post-prandial blood glucose" OR "diabetes mellitus type 2" OR "diabetes type 2" OR "type II diabetes mellitus" OR "type 2 diabetes" OR "type II diabetes" OR "advanced glycation end products" OR "HbA1c" OR "human hemoglobin A1c protein" OR "glycohemoglobin A" OR "glycosylated analysis hemoglobin A" OR "glycated hemoglobins" OR "glycated hemoglobin" OR "blood glucose metabolism" OR "blood glucose analysis") AND ("local" OR "subgingival" OR "subgingivally delivered" OR "subgingival delivery" OR "subgingival application" OR "locally delivered" OR "local use" OR "gel" OR "topical" OR "delivery vehicle" OR "nanospheres") AND ("antibiotic" OR "antibiotics" OR "antimicrobial" OR "antimicrobials" OR "anti-bacterial" OR "agents" OR "anti-bacterial agents" OR "antibacterial agents" OR "disinfection" OR "amoxicillin" OR "amox" OR "amoxicilline" OR "penicillin" OR "Azithromycin" OR "clarithromycin" OR "doxycycline" OR "doxycyclin" OR "Tetracycline" OR "minocycline" OR "clindamycin" OR "Fluoroquinolones" OR "metronidazole" OR "Chlorhexidine" OR "triclosan" OR "Cetylpyridinium" OR "Cetylpyridinium chloride" OR "CPC" OR "povidone iodine") AND ("Chronic Periodontitis" OR "Chronic Periodontitides" OR "Adult Periodontitis" OR "Adult Periodontitides" OR "Periodontal treatment" OR "Periodontal therapy" OR "nonsurgical periodontal therapy" OR "non-surgical periodontal therapy" OR "Periodontitis" OR "Periodontitides" OR "Periodontal Diseases" OR "Periodontal Disease" OR "Parodontosis" OR "Parodontoses" OR "Pyorrhea Alveolaris" OR "scaling and root planing" OR "Periodontal Pocket" OR "periodontal pockets" OR "pocket depth" OR "pocket probing" OR "probing depth" OR "periodontal health" OR "Alveolar Bone Loss" OR "Alveolar Bone Losses" OR "Alveolar Resorption" OR "Periodontal Bone Losses" OR "Periodontal Bone Loss" OR "Periodontal Resorption" OR "phase 1 periodontal therapy" OR "initial periodontal therapy" OR "Periodontal Index" OR "Periodontal Indices" OR "Periodontal Indexes" OR "bleeding on probing" OR "bleeding index" OR "periodontal inflammation" OR "Gingival Bleeding on Probing" OR "gingival bleeding" OR "Gingival Index" OR "Gingival Indices" OR "Gingival Indexes" OR "Dental Plaque Index" OR "Dental Plaque Indexes" OR "Dental Plaque Indices" OR "Dental Plaque" OR "dental biofilm" OR "dental biofilms" OR "Periodontal probing depth" OR "Clinical attachment loss" OR "Periodontal Debridement" OR "Periodontal Debridements" OR "Periodontal Pocket Debridement" OR "periodontal therapy" OR "Dental Scaling" OR "Root Scaling" OR "Subgingival Scaling" OR "periodontal treatment" OR "periodontal therapy" OR "nonsurgical periodontal treatment" OR "non-surgical periodontal treatment" OR "non-surgical mechanical therapy" OR "Root Planing" OR "Root Planings" OR "periodontal attachment loss" OR "clinical attachment level" OR "attachment gain" OR "periodontal healing" OR "periodontal diseased sites" OR "periodontal parameters" OR "periodontal clinical parameters" OR "pocket closure" OR "pocket depth reduction" OR "residual pockets" OR "periodontal recession" OR "Gingival Recession" OR "Gingival Recessions" OR "periodontal re-instrumentation" OR "pocket re-instrumentation" OR "periodontal residual sites") | 1^st^ search 194  2^nd^ search  168 |
| **Scopus** | TITLE-ABS-KEY(("Diabetes Mellitus" OR "Type 2 Diabetes Mellitus" OR "Diabetes" OR "Metabolic Diseases" OR "Hyperglycemia" OR "Glucose Metabolism Disorders" OR "Metabolic Control" OR "Glycated Hemoglobin A" OR "Glycated Hemoglobin" OR "HbA1" OR "Glycosylated Hemoglobin A" OR "Blood Glucose" OR "Glycemic Index" OR "Fasting blood glucose" OR "Post-prandial blood glucose" OR "diabetes mellitus type 2" OR "diabetes type 2" OR "type II diabetes mellitus" OR "type 2 diabetes" OR "type II diabetes" OR "advanced glycation end products" OR "HbA1c" OR "human hemoglobin A1c protein" OR "glycohemoglobin A" OR "glycosylated analysis hemoglobin A" OR "glycated hemoglobins" OR "glycated hemoglobin" OR "blood glucose metabolism" OR "blood glucose analysis") AND ("local" OR "subgingival" OR "subgingivally delivered" OR "subgingival delivery" OR "subgingival application" OR "locally delivered" OR "local use" OR "gel" OR "topical" OR "delivery vehicle" OR "nanospheres") AND ("antibiotic" OR "antibiotics" OR "antimicrobial" OR "antimicrobials" OR "anti-bacterial" OR "agents" OR "anti-bacterial agents" OR "antibacterial agents" OR "disinfection" OR "amoxicillin" OR "amox" OR "amoxicilline" OR "penicillin" OR "Azithromycin" OR "clarithromycin" OR "doxycycline" OR "doxycyclin" OR "Tetracycline" OR "minocycline" OR "clindamycin" OR "Fluoroquinolones" OR "metronidazole" OR "Chlorhexidine" OR "triclosan" OR "Cetylpyridinium" OR "Cetylpyridinium chloride" OR "CPC" OR "povidone iodine") AND ("Chronic Periodontitis" OR "Chronic Periodontitides" OR "Adult Periodontitis" OR "Adult Periodontitides" OR "Periodontal treatment" OR "Periodontal therapy" OR "nonsurgical periodontal therapy" OR "non-surgical periodontal therapy" OR "Periodontitis" OR "Periodontitides" OR "Periodontal Diseases" OR "Periodontal Disease" OR "Parodontosis" OR "Parodontoses" OR "Pyorrhea Alveolaris" OR "scaling and root planing" OR "Periodontal Pocket" OR "periodontal pockets" OR "pocket depth" OR "pocket probing" OR "probing depth" OR "periodontal health" OR "Alveolar Bone Loss" OR "Alveolar Bone Losses" OR "Alveolar Resorption" OR "Periodontal Bone Losses" OR "Periodontal Bone Loss" OR "Periodontal Resorption" OR "phase 1 periodontal therapy" OR "initial periodontal therapy" OR "Periodontal Index" OR "Periodontal Indices" OR "Periodontal Indexes" OR "bleeding on probing" OR "bleeding index" OR "periodontal inflammation" OR "Gingival Bleeding on Probing" OR "gingival bleeding" OR "Gingival Index" OR "Gingival Indices" OR "Gingival Indexes" OR "Dental Plaque Index" OR "Dental Plaque Indexes" OR "Dental Plaque Indices" OR "Dental Plaque" OR "dental biofilm" OR "dental biofilms" OR "Periodontal probing depth" OR "Clinical attachment loss" OR "Periodontal Debridement" OR "Periodontal Debridements" OR "Periodontal Pocket Debridement" OR "periodontal therapy" OR "Dental Scaling" OR "Root Scaling" OR "Subgingival Scaling" OR "periodontal treatment" OR "periodontal therapy" OR "nonsurgical periodontal treatment" OR "non-surgical periodontal treatment" OR "non-surgical mechanical therapy" OR "Root Planing" OR "Root Planings" OR "periodontal attachment loss" OR "clinical attachment level" OR "attachment gain" OR "periodontal healing" OR "periodontal diseased sites" OR "periodontal parameters" OR "periodontal clinical parameters" OR "pocket closure" OR "pocket depth reduction" OR "residual pockets" OR "periodontal recession" OR "Gingival Recession" OR "Gingival Recessions" OR "periodontal re-instrumentation" OR "pocket re-instrumentation" OR "periodontal residual sites")) | 1^st^ search 215  2^nd^ search 279 |
| **Web of Science** | ("Diabetes Mellitus" OR "Type 2 Diabetes Mellitus" OR "Diabetes" OR "Metabolic Diseases" OR "Hyperglycemia" OR "Glucose Metabolism Disorders" OR "Metabolic Control" OR "Glycated Hemoglobin A" OR "Glycated Hemoglobin" OR "HbA1" OR "Glycosylated Hemoglobin A" OR "Blood Glucose" OR "Glycemic Index" OR "Fasting blood glucose" OR "Post-prandial blood glucose" OR "diabetes mellitus type 2" OR "diabetes type 2" OR "type II diabetes mellitus" OR "type 2 diabetes" OR "type II diabetes" OR "advanced glycation end products" OR "HbA1c" OR "human hemoglobin A1c protein" OR "glycohemoglobin A" OR "glycosylated analysis hemoglobin A" OR "glycated hemoglobins" OR "glycated hemoglobin" OR "blood glucose metabolism" OR "blood glucose analysis") AND ("local" OR "subgingival" OR "subgingivally delivered" OR "subgingival delivery" OR "subgingival application" OR "locally delivered" OR "local use" OR "gel" OR "topical" OR "delivery vehicle" OR "nanospheres") AND ("antibiotic" OR "antibiotics" OR "antimicrobial" OR "antimicrobials" OR "anti-bacterial" OR "agents" OR "anti-bacterial agents" OR "antibacterial agents" OR "disinfection" OR "amoxicillin" OR "amox" OR "amoxicilline" OR "penicillin" OR "Azithromycin" OR "clarithromycin" OR "doxycycline" OR "doxycyclin" OR "Tetracycline" OR "minocycline" OR "clindamycin" OR "Fluoroquinolones" OR "metronidazole" OR "Chlorhexidine" OR "triclosan" OR "Cetylpyridinium" OR "Cetylpyridinium chloride" OR "CPC" OR "povidone iodine") AND ("Chronic Periodontitis" OR "Chronic Periodontitides" OR "Adult Periodontitis" OR "Adult Periodontitides" OR "Periodontal treatment" OR "Periodontal therapy" OR "nonsurgical periodontal therapy" OR "non-surgical periodontal therapy" OR "Periodontitis" OR "Periodontitides" OR "Periodontal Diseases" OR "Periodontal Disease" OR "Parodontosis" OR "Parodontoses" OR "Pyorrhea Alveolaris" OR "scaling and root planing" OR "Periodontal Pocket" OR "periodontal pockets" OR "pocket depth" OR "pocket probing" OR "probing depth" OR "periodontal health" OR "Alveolar Bone Loss" OR "Alveolar Bone Losses" OR "Alveolar Resorption" OR "Periodontal Bone Losses" OR "Periodontal Bone Loss" OR "Periodontal Resorption" OR "phase 1 periodontal therapy" OR "initial periodontal therapy" OR "Periodontal Index" OR "Periodontal Indices" OR "Periodontal Indexes" OR "bleeding on probing" OR "bleeding index" OR "periodontal inflammation" OR "Gingival Bleeding on Probing" OR "gingival bleeding" OR "Gingival Index" OR "Gingival Indices" OR "Gingival Indexes" OR "Dental Plaque Index" OR "Dental Plaque Indexes" OR "Dental Plaque Indices" OR "Dental Plaque" OR "dental biofilm" OR "dental biofilms" OR "Periodontal probing depth" OR "Clinical attachment loss" OR "Periodontal Debridement" OR "Periodontal Debridements" OR "Periodontal Pocket Debridement" OR "periodontal therapy" OR "Dental Scaling" OR "Root Scaling" OR "Subgingival Scaling" OR "periodontal treatment" OR "periodontal therapy" OR "nonsurgical periodontal treatment" OR "non-surgical periodontal treatment" OR "non-surgical mechanical therapy" OR "Root Planing" OR "Root Planings" OR "periodontal attachment loss" OR "clinical attachment level" OR "attachment gain" OR "periodontal healing" OR "periodontal diseased sites" OR "periodontal parameters" OR "periodontal clinical parameters" OR "pocket closure" OR "pocket depth reduction" OR "residual pockets" OR "periodontal recession" OR "Gingival Recession" OR "Gingival Recessions" OR "periodontal re-instrumentation" OR "pocket re-instrumentation" OR "periodontal residual sites") | 1^st^ search 78  2^nd^ search  107 |
| **LILACS (Portuguese and Spanish)** | ("Diabetes Mellitus" OR "Type 2 Diabetes Mellitus" OR "Diabetes" OR "Metabolic Diseases" OR "Hyperglycemia" OR "Glucose Metabolism Disorders" OR "Metabolic Control" OR "Glycated Hemoglobin A" OR "Glycated Hemoglobin" OR "HbA1" OR "Glycosylated Hemoglobin A" OR "Blood Glucose" OR "Glycemic Index" OR "Fasting blood glucose" OR "Post-prandial blood glucose" OR "diabetes mellitus type 2" OR "diabetes type 2" OR "type II diabetes mellitus" OR "type 2 diabetes" OR "type II diabetes" OR "advanced glycation end products" OR "HbA1c" OR "human hemoglobin A1c protein" OR "glycohemoglobin A" OR "glycosylated analysis hemoglobin A" OR "glycated hemoglobins" OR "glycated hemoglobin" OR "blood glucose metabolism" OR "blood glucose analysis" OR "Diabetes Mellitus" OR "Diabetes mellitus tipo 2" OR "doenças metabólicas" OR "enfermedades metabólicas" OR "Hiperglicemia" OR "hiperglucemia" OR "Tanstornos do metabolismo de glucose" OR "Transtornos del metabolismo de la glucosa" OR "Hemoglobina A Glicada" OR "Hemoglobina A Glucada" OR "glicemia" OR "glucemia" OR "índice glicêmico" OR "índice glucémico" OR "produtos finais de glicação avançada" OR "productos finales de glicación avanzada") AND ("local" OR "subgingival" OR "subgingivally delivered" OR "subgingival delivery" OR "subgingival application" OR "locally delivered" OR "local use" OR "gel" OR "topical" OR "delivery vehicle" OR "nanospheres" OR "subgengival" OR "subgingival" OR "Nanosferas") AND ("antibiotic" OR "antibiotics" OR "antimicrobial" OR "antimicrobials" OR "anti-bacterial" OR "agents" OR "anti-bacterial agents" OR "antibacterial agents" OR "disinfection" OR "amoxicillin" OR "amox" OR "amoxicilline" OR "penicillin" OR "Azithromycin" OR "clarithromycin" OR "doxycycline" OR "doxycyclin" OR "Tetracycline" OR "minocycline" OR "clindamycin" OR "Fluoroquinolones" OR "metronidazole" OR "Chlorhexidine" OR "triclosan" OR "Cetylpyridinium" OR "Cetylpyridinium chloride" OR "CPC" OR "povidone iodine" OR "antibacterianos" OR "Anti-Infecciosos" OR "Antimicrobianos" OR "Antiinfecciosos" OR "desinfecção" OR "desinfección" OR "Amoxicilina" OR "Penicilina" OR "Azitromicina" OR "Claritromicina" OR "Doxiciclina" OR "Tetraciclina" OR "Minociclina" OR "Clindamicina" OR "Fluoroquinolonas" OR "Metronidazol" OR "clorexidina" OR "clorhexidina" OR "triclosano" OR "triclosán" OR "cetilpiridínio" OR "cloreto de cetilpiridínio" OR "Cloruro de Cetilpiridino" OR "Povidona-Iodo" OR "Povidona Yodada") AND ("Chronic Periodontitis" OR "Chronic Periodontitides" OR "Adult Periodontitis" OR "Adult Periodontitides" OR "Periodontal treatment" OR "Periodontal therapy" OR "nonsurgical periodontal therapy" OR "non-surgical periodontal therapy" OR "Periodontitis" OR "Periodontitides" OR "Periodontal Diseases" OR "Periodontal Disease" OR "Parodontosis" OR "Parodontoses" OR "Pyorrhea Alveolaris" OR "scaling and root planing" OR "Periodontal Pocket" OR "periodontal pockets" OR "pocket depth" OR "pocket probing" OR "probing depth" OR "periodontal health" OR "Alveolar Bone Loss" OR "Alveolar Bone Losses" OR "Alveolar Resorption" OR "Periodontal Bone Losses" OR "Periodontal Bone Loss" OR "Periodontal Resorption" OR "phase 1 periodontal therapy" OR "initial periodontal therapy" OR "Periodontal Index" OR "Periodontal Indices" OR "Periodontal Indexes" OR "bleeding on probing" OR "bleeding index" OR "periodontal inflammation" OR "Gingival Bleeding on Probing" OR "gingival bleeding" OR "Gingival Index" OR "Gingival Indices" OR "Gingival Indexes" OR "Dental Plaque Index" OR "Dental Plaque Indexes" OR "Dental Plaque Indices" OR "Dental Plaque" OR "dental biofilm" OR "dental biofilms" OR "Periodontal probing depth" OR "Clinical attachment loss" OR "Periodontal Debridement" OR "Periodontal Debridements" OR "Periodontal Pocket Debridement" OR "periodontal therapy" OR "Dental Scaling" OR "Root Scaling" OR "Subgingival Scaling" OR "periodontal treatment" OR "periodontal therapy" OR "nonsurgical periodontal treatment" OR "non-surgical periodontal treatment" OR "non-surgical mechanical therapy" OR "Root Planing" OR "Root Planings" OR "periodontal attachment loss" OR "clinical attachment level" OR "attachment gain" OR "periodontal healing" OR "periodontal diseased sites" OR "periodontal parameters" OR "periodontal clinical parameters" OR "pocket closure" OR "pocket depth reduction" OR "residual pockets" OR "periodontal recession" OR "Gingival Recession" OR "Gingival Recessions" OR "periodontal re-instrumentation" OR "pocket re-instrumentation" OR "periodontal residual sites" OR "Periodontite crônica" OR "periodontitis crónica" OR "desbridamento periodontal" OR "Desbridamiento Periodontal" OR "aplainamento radicular" OR "aplanamiento de la raíz" OR "raspagem dentária" OR "raspado dental" OR "curetagem subgengival" OR "curetaje subgingival" OR "Periodontite" OR "Periodontitis" OR "doenças periodontais" OR "enfermedades periodontales" OR "bolsa periodontal" OR "índice periodontal" OR "Índice de Placa Dentária" OR "Índice de Placa Dental" OR "Placa Dentária" OR "Placa Dental" OR "perda da inserção periodontal" OR "pérdida de la inserción periodontal" OR "Retração Gengival" OR "Recesión Gingival") | 1^st^ search 19  2^nd^ search  19 |
| **ProQuest** | noft(("Diabetes Mellitus" OR "Type 2 Diabetes Mellitus" OR "Diabetes" OR "Metabolic Diseases" OR "Hyperglycemia" OR "Glucose Metabolism Disorders" OR "Metabolic Control" OR "Glycated Hemoglobin A" OR "Glycated Hemoglobin" OR "HbA1" OR "Glycosylated Hemoglobin A" OR "Blood Glucose" OR "Glycemic Index" OR "Fasting blood glucose" OR "Post-prandial blood glucose" OR "diabetes mellitus type 2" OR "diabetes type 2" OR "type II diabetes mellitus" OR "type 2 diabetes" OR "type II diabetes" OR "advanced glycation end products" OR "HbA1c" OR "human hemoglobin A1c protein" OR "glycohemoglobin A" OR "glycosylated analysis hemoglobin A" OR "glycated hemoglobins" OR "glycated hemoglobin" OR "blood glucose metabolism" OR "blood glucose analysis") AND ("local" OR "subgingival" OR "subgingivally delivered" OR "subgingival delivery" OR "subgingival application" OR "locally delivered" OR "local use" OR "gel" OR "topical" OR "delivery vehicle" OR "nanospheres") AND ("antibiotic" OR "antibiotics" OR "antimicrobial" OR "antimicrobials" OR "anti-bacterial" OR "agents" OR "anti-bacterial agents" OR "antibacterial agents" OR "disinfection" OR "amoxicillin" OR "amox" OR "amoxicilline" OR "penicillin" OR "Azithromycin" OR "clarithromycin" OR "doxycycline" OR "doxycyclin" OR "Tetracycline" OR "minocycline" OR "clindamycin" OR "Fluoroquinolones" OR "metronidazole" OR "Chlorhexidine" OR "triclosan" OR "Cetylpyridinium" OR "Cetylpyridinium chloride" OR "CPC" OR "povidone iodine") AND ("Chronic Periodontitis" OR "Chronic Periodontitides" OR "Adult Periodontitis" OR "Adult Periodontitides" OR "Periodontal treatment" OR "Periodontal therapy" OR "nonsurgical periodontal therapy" OR "non-surgical periodontal therapy" OR "Periodontitis" OR "Periodontitides" OR "Periodontal Diseases" OR "Periodontal Disease" OR "Parodontosis" OR "Parodontoses" OR "Pyorrhea Alveolaris" OR "scaling and root planing" OR "Periodontal Pocket" OR "periodontal pockets" OR "pocket depth" OR "pocket probing" OR "probing depth" OR "periodontal health" OR "Alveolar Bone Loss" OR "Alveolar Bone Losses" OR "Alveolar Resorption" OR "Periodontal Bone Losses" OR "Periodontal Bone Loss" OR "Periodontal Resorption" OR "phase 1 periodontal therapy" OR "initial periodontal therapy" OR "Periodontal Index" OR "Periodontal Indices" OR "Periodontal Indexes" OR "bleeding on probing" OR "bleeding index" OR "periodontal inflammation" OR "Gingival Bleeding on Probing" OR "gingival bleeding" OR "Gingival Index" OR "Gingival Indices" OR "Gingival Indexes" OR "Dental Plaque Index" OR "Dental Plaque Indexes" OR "Dental Plaque Indices" OR "Dental Plaque" OR "dental biofilm" OR "dental biofilms" OR "Periodontal probing depth" OR "Clinical attachment loss" OR "Periodontal Debridement" OR "Periodontal Debridements" OR "Periodontal Pocket Debridement" OR "periodontal therapy" OR "Dental Scaling" OR "Root Scaling" OR "Subgingival Scaling" OR "periodontal treatment" OR "periodontal therapy" OR "nonsurgical periodontal treatment" OR "non-surgical periodontal treatment" OR "non-surgical mechanical therapy" OR "Root Planing" OR "Root Planings" OR "periodontal attachment loss" OR "clinical attachment level" OR "attachment gain" OR "periodontal healing" OR "periodontal diseased sites" OR "periodontal parameters" OR "periodontal clinical parameters" OR "pocket closure" OR "pocket depth reduction" OR "residual pockets" OR "periodontal recession" OR "Gingival Recession" OR "Gingival Recessions" OR "periodontal re-instrumentation" OR "pocket re-instrumentation" OR "periodontal residual sites")) | 1^st^ search 12  2^nd^ search 63 |
| **Open Grey** | ("Diabetes Mellitus" OR "Type 2 Diabetes Mellitus" OR "Diabetes" OR "Metabolic Diseases" OR "Hyperglycemia" OR "Glucose Metabolism Disorders" OR "Metabolic Control" OR "Glycated Hemoglobin A" OR "Glycated Hemoglobin" OR "HbA1" OR "Glycosylated Hemoglobin A" OR "Blood Glucose" OR "Glycemic Index" OR "Fasting blood glucose" OR "Post-prandial blood glucose" OR "diabetes mellitus type 2" OR "diabetes type 2" OR "type II diabetes mellitus" OR "type 2 diabetes" OR "type II diabetes" OR "advanced glycation end products" OR "HbA1c" OR "human hemoglobin A1c protein" OR "glycohemoglobin A" OR "glycosylated analysis hemoglobin A" OR "glycated hemoglobins" OR "glycated hemoglobin" OR "blood glucose metabolism" OR "blood glucose analysis") AND ("local" OR "subgingival" OR "subgingivally delivered" OR "subgingival delivery" OR "subgingival application" OR "locally delivered" OR "local use" OR "gel" OR "topical" OR "delivery vehicle" OR "nanospheres") AND ("antibiotic" OR "antibiotics" OR "antimicrobial" OR "antimicrobials" OR "anti-bacterial" OR "agents" OR "anti-bacterial agents" OR "antibacterial agents" OR "disinfection" OR "amoxicillin" OR "amox" OR "amoxicilline" OR "penicillin" OR "Azithromycin" OR "clarithromycin" OR "doxycycline" OR "doxycyclin" OR "Tetracycline" OR "minocycline" OR "clindamycin" OR "Fluoroquinolones" OR "metronidazole" OR "Chlorhexidine" OR "triclosan" OR "Cetylpyridinium" OR "Cetylpyridinium chloride" OR "CPC" OR "povidone iodine") AND ("Chronic Periodontitis" OR "Chronic Periodontitides" OR "Adult Periodontitis" OR "Adult Periodontitides" OR "Periodontal treatment" OR "Periodontal therapy" OR "nonsurgical periodontal therapy" OR "non-surgical periodontal therapy" OR "Periodontitis" OR "Periodontitides" OR "Periodontal Diseases" OR "Periodontal Disease" OR "Parodontosis" OR "Parodontoses" OR "Pyorrhea Alveolaris" OR "scaling and root planing" OR "Periodontal Pocket" OR "periodontal pockets" OR "pocket depth" OR "pocket probing" OR "probing depth" OR "periodontal health" OR "Alveolar Bone Loss" OR "Alveolar Bone Losses" OR "Alveolar Resorption" OR "Periodontal Bone Losses" OR "Periodontal Bone Loss" OR "Periodontal Resorption" OR "phase 1 periodontal therapy" OR "initial periodontal therapy" OR "Periodontal Index" OR "Periodontal Indices" OR "Periodontal Indexes" OR "bleeding on probing" OR "bleeding index" OR "periodontal inflammation" OR "Gingival Bleeding on Probing" OR "gingival bleeding" OR "Gingival Index" OR "Gingival Indices" OR "Gingival Indexes" OR "Dental Plaque Index" OR "Dental Plaque Indexes" OR "Dental Plaque Indices" OR "Dental Plaque" OR "dental biofilm" OR "dental biofilms" OR "Periodontal probing depth" OR "Clinical attachment loss" OR "Periodontal Debridement" OR "Periodontal Debridements" OR "Periodontal Pocket Debridement" OR "periodontal therapy" OR "Dental Scaling" OR "Root Scaling" OR "Subgingival Scaling" OR "periodontal treatment" OR "periodontal therapy" OR "nonsurgical periodontal treatment" OR "non-surgical periodontal treatment" OR "non-surgical mechanical therapy" OR "Root Planing" OR "Root Planings" OR "periodontal attachment loss" OR "clinical attachment level" OR "attachment gain" OR "periodontal healing" OR "periodontal diseased sites" OR "periodontal parameters" OR "periodontal clinical parameters" OR "pocket closure" OR "pocket depth reduction" OR "residual pockets" OR "periodontal recession" OR "Gingival Recession" OR "Gingival Recessions" OR "periodontal re-instrumentation" OR "pocket re-instrumentation" OR "periodontal residual sites") | 1^st^ search  0  2^nd^ search  Unavailable |
| **Google Scholar** | ("Type 2 Diabetes mellitus" OR "diabetes mellitus") AND ("periodontitis" OR "chronic periodontitis") AND ("non-surgical periodontal therapy" OR "scaling and root planing") AND ("antimicrobials") AND ("local" OR "subgingival") AND ("HbA1c") | 1^st^ search 210  2^nd^ search 341 |
| **Clinical Trial registry** | Condition or disease: Periodontitis  Other terms: Antimicrobials AND diabetes | 1^st^ search 20  2^nd^ search 27 |
| **ICTRP WHO** | periodontitis AND diabetes AND antimicrobials | 1^st^ search  0  2^nd^ search  0 |
