# Supplementary material for: Efficacy of Adjunctive Local Antimicrobials to Non-Surgical Periodontal Therapy in Pocket Reduction and Glycemic Control of Patients with Type 2 Diabetes: A Network Meta-Analysis
Source: Curr Diabetes Rev. 2024 Sep 3;21(7):E15733998320667. doi: 10.2174/0115733998320667240805045742 (PMC12082567; doi:10.2174/0115733998320667240805045742)
Supplement: Supplementary file 1 [file CDR-21-7-E15733998320667_SD1.zip › CDR-21-7-E15733998320667_SD1/S3 - League table for each comparison Mean Differences.docx]

**Supplementary file 3: League table showing the effect estimates (in Mean Difference) and 95%CI for each comparison.**

The lower triangle shows the results of mixed comparisons; the upper triangle shows direct comparisons. Negative results favor treatments showed in the columns, positive results favor treatment in the rows. Bold results indicate that the treatment in the column was more effective compared to the intervention in the line. The colors show the certainty of evidence for each comparison, according to the legend on the lower left (green: high certainty; yellow: moderate certainty; orange: low certainty; light red: very low certainty).

**Comparisons for HbA1c at 3 months**

| Doxy | . | . | . | . | -0.80 [-1.70; 0.10] |
| --- | --- | --- | --- | --- | --- |
| -0.12 [-1.24; 1.00] | CHX_Gel | . | . | . | -0.68 [-1.34; -0.02] |
| -0.18 [-1.11; 0.75] | -0.06 [-0.75; 0.64] | Tetra_Fyber | . | . | -0.62 [-0.85; -0.39] |
| -0.61 [-1.74; 0.52] | -0.49 [-1.43; 0.46] | -0.43 [-1.14; 0.28] | Tetra_Oint | . | -0.19 [-0.87; 0.49] |
| -0.69 [-1.98; 0.59] | -0.57 [-1.70; 0.55] | -0.51 [-1.45; 0.43] | -0.08 [-1.22; 1.05] | Mino | -0.11 [-1.02; 0.80] |
| -0.80 [-1.70; 0.10] | -0.68 [-1.34; -0.02] | -0.62 [-0.85; -0.39] | -0.19 [-0.87; 0.49] | -0.11 [-1.02; 0.80] | Control (NSPT) |

**Comparisons for HbA1c at 6 months**

| CHX_Gel | . | . | -0.53 [-1.57; 0.51] |
| --- | --- | --- | --- |
| -0.47 [-2.86; 1.92] | Mino | . | -0.06 [-2.21; 2.09] |
| -0.53 [-2.31; 1.24] | -0.06 [-2.65; 2.53] | Doxy | -0.00 [-1.44; 1.44] |
| -0.53 [-1.57; 0.51] | -0.06 [-2.21; 2.09] | 0.00 [-1.44; 1.44] | Control (NSPT) |

**Comparisons for PPD at 3 months**

| STZ | . | . | . | . | . | -1.30 [-2.22; -0.38] | . |
| --- | --- | --- | --- | --- | --- | --- | --- |
| -0.29 [-1.34; 0.76] | CLM | . | . | . | . | -1.01 [-1.50; -0.52] | . |
| -0.38 [-1.56; 0.80] | -0.09 [-0.97; 0.79] | Tetra_Fiber | . | . | . | -0.92 [-1.66; -0.18] | . |
| -1.04 [-2.15; 0.07] | -0.75 [-1.53; 0.03] | -0.66 [-1.61; 0.29] | AZT | . | . | -0.26 [-0.87; 0.35] | . |
| -1.30 [-2.37; -0.23] | -1.01 [-1.74; -0.28] | -0.92 [-1.83; -0.01] | -0.26 [-1.07; 0.55] | Doxy | . | -0.00 [-0.54; 0.54] | . |
| -1.30 [-2.30; -0.30] | -1.01 [-1.63; -0.39] | -0.92 [-1.75; -0.09] | -0.26 [-0.98; 0.46] | -0.00 [-0.67; 0.66] | CHX_Gel | 0.00 [-0.38; 0.38] | . |
| -1.30 [-2.22; -0.38] | -1.01 [-1.50; -0.52] | -0.92 [-1.66; -0.18] | -0.26 [-0.87; 0.35] | 0.00 [-0.54; 0.54] | 0.00 [-0.38; 0.38] | Control (NSPT) | -0.05 [-0.91; 0.81] |
| -1.35 [-2.61; -0.09] | -1.06 [-2.05; -0.07] | -0.97 [-2.10; 0.16] | -0.31 [-1.36; 0.74] | -0.05 [-1.06; 0.96] | -0.05 [-0.99; 0.89] | -0.05 [-0.91; 0.81] | Mino |

**Comparisons for PPD at 6 months**

| STZ | . | . | . | -2.64 [-3.56; -1.72] | . | . |
| --- | --- | --- | --- | --- | --- | --- |
| -1.66 [-2.72; -0.60] | CLM | . | . | -0.98 [-1.51; -0.45] | . | . |
| -2.41 [-3.47; -1.35] | -0.75 [-1.51; 0.01] | AZT | . | -0.23 [-0.77; 0.31] | . | . |
| -2.64 [-3.91; -1.37] | -0.98 [-2.00; 0.04] | -0.23 [-1.26; 0.80] | Mino | -0.00 [-0.87; 0.87] | . | . |
| -2.64 [-3.56; -1.72] | -0.98 [-1.51; -0.45] | -0.23 [-0.77; 0.31] | 0.00 [-0.87; 0.87] | Control (NSPT) | -0.10 [-0.67; 0.47] | -0.09 [-0.48; 0.30] |
| -2.74 [-3.82; -1.66] | -1.08 [-1.86; -0.30] | -0.33 [-1.12; 0.46] | -0.10 [-1.14; 0.94] | -0.10 [-0.67; 0.47] | Doxy | . |
| -2.73 [-3.72; -1.73] | -1.07 [-1.73; -0.41] | -0.32 [-0.98; 0.35] | -0.09 [-1.05; 0.87] | -0.09 [-0.48; 0.30] | 0.01 [-0.68; 0.70] | CHX_Gel |

**Certainty of Evidence**

| High |
| --- |
| Moderate |
| Low |
| Very Low |
