# Supplementary material for: Efficacy of Adjunctive Local Antimicrobials to Non-Surgical Periodontal Therapy in Pocket Reduction and Glycemic Control of Patients with Type 2 Diabetes: A Network Meta-Analysis
Source: Curr Diabetes Rev. 2024 Sep 3;21(7):E15733998320667. doi: 10.2174/0115733998320667240805045742 (PMC12082567; doi:10.2174/0115733998320667240805045742)
Supplement: Supplementary file 1 [file CDR-21-7-E15733998320667_SD1.zip › CDR-21-7-E15733998320667_SD1/S4 - Assessment of inconsistency for all studies (MD).docx]

**Supplementary File S4: Assessment of inconsistency for all studies (MD)**

**Results NMA HbA1c 3 months**

| **Comparison** | **N. Studies** | **NMA** | **Direct** | **Indirect** | **Difference** | **Diff_95CI_lower** | **Diff_95CI_upper** | **p-Value** |
| --- | --- | --- | --- | --- | --- | --- | --- | --- |
| CHX_Gel:Control | 2 | -0.68 | -0.68 | NA | NA | NA | NA | NA |
| CHX_Gel:Doxy | 0 | 0.12 | NA | 0.12 | NA | NA | NA | NA |
| CHX_Gel:Mino | 0 | -0.57 | NA | -0.57 | NA | NA | NA | NA |
| CHX_Gel:Tetra_Fyber | 0 | -0.06 | NA | -0.06 | NA | NA | NA | NA |
| CHX_Gel:Tetra_Oint | 0 | -0.49 | NA | -0.49 | NA | NA | NA | NA |
| Doxy:Control | 1 | -0.80 | -0.80 | NA | NA | NA | NA | NA |
| Mino:Control | 2 | -0.11 | -0.11 | NA | NA | NA | NA | NA |
| Tetra_Fyber:Control | 1 | -0.62 | -0.62 | NA | NA | NA | NA | NA |
| Tetra_Oint:Control | 1 | -0.19 | -0.19 | NA | NA | NA | NA | NA |
| Doxy:Mino | 0 | -0.69 | NA | -0.69 | NA | NA | NA | NA |
| Doxy:Tetra_Fyber | 0 | -0.18 | NA | -0.18 | NA | NA | NA | NA |
| Doxy:Tetra_Oint | 0 | -0.61 | NA | -0.61 | NA | NA | NA | NA |
| Mino:Tetra_Fyber | 0 | 0.51 | NA | 0.51 | NA | NA | NA | NA |
| Mino:Tetra_Oint | 0 | 0.08 | NA | 0.08 | NA | NA | NA | NA |
| Tetra_Fyber: Tetra_Oint | 0 | -0.43 | NA | -0.43 | NA | NA | NA | NA |

**Results NMA HbA1c 6 months**

| **Comparison** | **N. Studies** | **NMA** | **Direct** | **Indirect** | **Difference** | **Diff_95CI_lower** | **Diff_95CI_upper** | **p-Value** |
| --- | --- | --- | --- | --- | --- | --- | --- | --- |
| CHX_Gel:Control | 2 | -0.53 | -0.53 | NA | NA | NA | NA | NA |
| CHX_Gel:Doxy | 0 | -0.53 | NA | -0.53 | NA | NA | NA | NA |
| CHX_Gel:Mino | 0 | -0.47 | NA | -0.47 | NA | NA | NA | NA |
| Doxy:Control | 1 | 0.00 | -0.00 | NA | NA | NA | NA | NA |
| Mino:Control | 1 | -0.06 | -0.06 | NA | NA | NA | NA | NA |
| Doxy:Mino | 0 | 0.06 | NA | 0.06 | NA | NA | NA | NA |

**Results NMA PPD 3 months**

| **Comparison** | **N. Studies** | **NMA** | **Direct** | **Indirect** | **Difference** | **Diff_95CI_lower** | **Diff_95CI_upper** | **p-Value** |
| --- | --- | --- | --- | --- | --- | --- | --- | --- |
| AZT:CHX_Gel | 0 | -0.26 | NA | -0.26 | NA | NA | NA | NA |
| AZT:CLM | 0 | 0.75 | NA | 0.75 | NA | NA | NA | NA |
| AZT:Control | 1 | -0.26 | -0.26 | NA | NA | NA | NA | NA |
| AZT:Doxy | 0 | -0.26 | NA | -0.26 | NA | NA | NA | NA |
| AZT:Mino | 0 | -0.31 | NA | -0.31 | NA | NA | NA | NA |
| AZT:STZ | 0 | 1.04 | NA | 1.04 | NA | NA | NA | NA |
| AZT:Tetra_Fiber | 0 | 0.66 | NA | 0.66 | NA | NA | NA | NA |
| CHX_Gel:CLM | 0 | 1.01 | NA | 1.01 | NA | NA | NA | NA |
| CHX_Gel:Control | 2 | 0.00 | 0.00 | NA | NA | NA | NA | NA |
| CHX_Gel:Doxy | 0 | 0.00 | NA | 0.00 | NA | NA | NA | NA |
| CHX_Gel:Mino | 0 | -0.05 | NA | -0.05 | NA | NA | NA | NA |
| CHX_Gel:STZ | 0 | 1.30 | NA | 1.30 | NA | NA | NA | NA |
| CHX_Gel:Tetra_Fiber | 0 | 0.92 | NA | 0.92 | NA | NA | NA | NA |
| CLM:Control | 1 | -1.01 | -1.01 | NA | NA | NA | NA | NA |
| CLM:Doxy | 0 | -1.01 | NA | -1.01 | NA | NA | NA | NA |
| CLM:Mino | 0 | -1.06 | NA | -1.06 | NA | NA | NA | NA |
| CLM:STZ | 0 | 0.29 | NA | 0.29 | NA | NA | NA | NA |
| CLM:Tetra_Fiber | 0 | -0.09 | NA | -0.09 | NA | NA | NA | NA |
| Doxy:Control | 1 | 0.00 | -0.00 | NA | NA | NA | NA | NA |
| Mino:Control | 1 | 0.05 | 0.05 | NA | NA | NA | NA | NA |
| STZ:Control | 1 | -1.30 | -1.30 | NA | NA | NA | NA | NA |
| Tetra_Fiber:Control | 1 | -0.92 | -0.92 | NA | NA | NA | NA | NA |
| Doxy:Mino | 0 | -0.05 | NA | -0.05 | NA | NA | NA | NA |
| Doxy:STZ | 0 | 1.30 | NA | 1.30 | NA | NA | NA | NA |
| Doxy:Tetra_Fiber | 0 | 0.92 | NA | 0.92 | NA | NA | NA | NA |
| Mino:STZ | 0 | 1.35 | NA | 1.35 | NA | NA | NA | NA |
| Mino:Tetra_Fiber | 0 | 0.97 | NA | 0.97 | NA | NA | NA | NA |
| STZ:Tetra_Fiber | 0 | -0.38 | NA | -0.38 | NA | NA | NA | NA |

**Results NMA PPD 6 months**

| **Comparison** | **N. Studies** | **NMA** | **Direct** | **Indirect** | **Difference** | **Diff_95CI_lower** | **Diff_95CI_upper** | **p-Value** |
| --- | --- | --- | --- | --- | --- | --- | --- | --- |
| AZT:CHX_Gel | 0 | -0.32 | NA | -0.32 | NA | NA | NA | NA |
| AZT:CLM | 0 | 0.75 | NA | 0.75 | NA | NA | NA | NA |
| AZT:Control | 1 | -0.23 | -0.23 | NA | NA | NA | NA | NA |
| AZT:Doxy | 0 | -0.33 | NA | -0.33 | NA | NA | NA | NA |
| AZT:Mino | 0 | -0.23 | NA | -0.23 | NA | NA | NA | NA |
| AZT:STZ | 0 | 2.41 | NA | 2.41 | NA | NA | NA | NA |
| CHX_Gel:CLM | 0 | 1.07 | NA | 1.07 | NA | NA | NA | NA |
| CHX_Gel:Control | 2 | 0.09 | 0.09 | NA | NA | NA | NA | NA |
| CHX_Gel:Doxy | 0 | -0.01 | NA | -0.01 | NA | NA | NA | NA |
| CHX_Gel:Mino | 0 | 0.09 | NA | 0.09 | NA | NA | NA | NA |
| CHX_Gel:STZ | 0 | 2.73 | NA | 2.73 | NA | NA | NA | NA |
| CLM:Control | 1 | -0.98 | -0.98 | NA | NA | NA | NA | NA |
| CLM:Doxy | 0 | -1.08 | NA | -1.08 | NA | NA | NA | NA |
| CLM:Mino | 0 | -0.98 | NA | -0.98 | NA | NA | NA | NA |
| CLM:STZ | 0 | 1.66 | NA | 1.66 | NA | NA | NA | NA |
| Doxy:Control | 1 | 0.10 | 0.10 | NA | NA | NA | NA | NA |
| Mino:Control | 1 | 0.00 | -0.00 | NA | NA | NA | NA | NA |
| STZ:Control | 1 | -2.64 | -2.64 | NA | NA | NA | NA | NA |
| Doxy:Mino | 0 | 0.10 | NA | 0.10 | NA | NA | NA | NA |
| Doxy:STZ | 0 | 2.74 | NA | 2.74 | NA | NA | NA | NA |
| Mino:STZ | 0 | 2.64 | NA | 2.64 | NA | NA | NA | NA |
