# Supplementary material for: Efficacy of Adjunctive Local Antimicrobials to Non-Surgical Periodontal Therapy in Pocket Reduction and Glycemic Control of Patients with Type 2 Diabetes: A Network Meta-Analysis
Source: Curr Diabetes Rev. 2024 Sep 3;21(7):E15733998320667. doi: 10.2174/0115733998320667240805045742 (PMC12082567; doi:10.2174/0115733998320667240805045742)
Supplement: Supplementary file 1 [file CDR-21-7-E15733998320667_SD1.zip › CDR-21-7-E15733998320667_SD1/S5 - GRADE explanations and results.docx]

**Supplementary file 5: GRADE explanations and results**

**GRADE Approach result for HbA1c at 3 months**

Criteria used to assess certainty of evidence was based on the Cochrane Training - [https://training.cochrane.org/resource/grade-approach-rate-certainty-evidence-network-meta-analysis-and-summary-findings-tables](about:blank) and apropos literature (listed below).

| **Criteria** | **Rated down direct estimate if:** | **Analysis** |
| --- | --- | --- |
| Risk of bias | When at least one study on the direct comparison presented high risk or some concerns due to problems in the randomization process and lack of blinding. | We rated down the certainty in one level due to some concerns and two levels due to high risk of bias in the randomization process.  On the other hand, since glycate hemoglobin is assessed through an automatic laboratory process, no problems with blinding were considered and we did not rate down the certainty. |
| Inconsistency | - If effect estimates were similar across studies (Guyatt et al. 2011b); - Overlap of 95%Cl (Guyatt et al. 2011b); - I^2^ for direct comparisons if was either moderate (30-60%), substantial (50-90%) or considerable (75%-100%) (Schunemann et al., 2021). - If only one study contributed to the direct effect estimate, the certainty was not rated down (Guyatt et al. 2011b). | We did not rate down the certainty for single studies forming the effect estimate.  Two comparisons had 2 studies included (CHX_Gel vs NSPT – Faramarzi et al., 2017 and Santos et al., 2013; Mino vs NSPT – Lin et al., 2012 and Matsumoto et al., 2009), but for these we did not downgrade the level of certainty of evidence because the confidence intervals overlapped and there was no inconsistency in the direction of the effect estimate. |
| Indirectness | Rate down if more than 30% of weight of pooled estimate came from studies which the populations had different characteristics (age, healthy condition) compared to the population of the PICO question (Brignardello-Petersen et al. 2018a). | We assessed indirectness for each comparison. For almost all comparisons, patients had similar characteristics regarding age (late adulthood/elders), T2D and the presence of periodontitis. All patients met the periodontitis case-definition, according to the New Periodontal Diseases Classification (Tonetti et al., 2018). Thus, there was no problems regarding periodontitis condition.  The initial glycated hemoglobin (HbA1c) was similar in all populations at the beginning of the study (ranging from 7.1 to 10.4%). The included patients had moderate to unsatisfactory metabolic control. |
| Publication bias | If industry funding was associated with a positive outcome favoring the sponsored treatment (Martins et al. 2019). | Of the studies included in this network meta-analysis,  only 1 received funded by the industry (DOXY vs NSPT – Lecio et al., 2019). However, although the results favored the treated group, there was no statistical difference for HbA1c in the study. Thus, the level was not downgraded due to publication bias. |
| **Criteria** | **Rated down indirect estimate if:** | **Analysis** |
| Intransitivity | If there were differences in study characteristics that may modify treatment effect in the direct comparisons that form the basis for the indirect estimate; and if those differences were regarding population, intervention, comparison, or outcome (Puhan et al. 2014). | For intransitivity, we considered the most dominant first order loop, or, in its absence, the closest direct comparison contributing to the effect estimate. Whenever the comparisons contributing to indirect evidence showed important modifications on population or glycated hemoglobin, one point was downgraded due to intransitivity. |
| **Criteria** | **Rated down NMA estimate if:** | **Analysis** |
| Incoherence | If node splitting test showed p-value <0.05, and direct, indirect and NMA evidence agreed with each other. (Brignardello-Petersen et al. 2018a). | Node split test was not feasible, due to the poorly connected network. In addition, it was not possible to compare the coherence between the direct and indirect estimates of the network meta-analysis. Thus, the certainty of evidence was not downgraded due to incoherence. |
| Imprecision | A partially contextualized approach was used, considering the large effect threshold according to Cohen’s classification (>0.8 SD) (Guyatt et al. 2011a; Schünemann et al. 2021; Brignardello-Petersen et al. 2021). | We downgraded in one level if the effect estimate crossed the minimum important difference (MID), considered as 0.5%; two levels if the confidence interval crossed both the MID and the null effect line.  In the cases when the threshold was not crossed, the effect size was evaluated. OIS calculations were performed to large unplausible effects. Whenever OIS was not achieved, one level was downgraded (Brignardello-Petersen et al. 2021). |

**Table S1: Assessment of GRADE for direct and indirect comparisons evidence certainty for HbA1c (3 months comparisons).**

| **Comparison** | **Number of**  **Studies** | **Risk of Bias** | **Inconsistency** | **Indirectness** | **Publication Bias** | **Direct comparison Certainty** | **Indirect evidence comparison from the closest loop** | **Indirect evidence comparison from the closest loop** | **Lowest between both** | **Intransitivity** | **Incoherence for NMA** | **Imprecision for NMA** | **Final Certainty** |
| --- | --- | --- | --- | --- | --- | --- | --- | --- | --- | --- | --- | --- | --- |
| **DIRECT COMPARISONS** | | | | | | | | | | | | | |
| CHX_Gel vs NSPT | 2 | -1 (some conc) | 0 | 0 | 0 | Moderate | - | - | - | - | - | -1 | Low |
| Doxy vs NSPT | 1 | 0 | 0 | 0 | 0 | High | - | - | - | - | - | -2 | Low |
| Mino vs NSPT | 2 | -1 (some conc) | 0 | 0 | 0 | Moderate | - | - | - | - | - | -2 | Very low |
| Tetra_Fyber vs NSPT | 1 | -1 (some conc) | 0 | 0 | 0 | Moderate | - | - | - | - | - | -1 | Low |
| Tetra_Oint vs NSPT | 1 | -1 (some conc) | 0 | 0 | 0 | Moderate | - | - | - | - | - | -2 | Very low |
| **INDIRECT ONLY COMPARISONS** | | | | | | | | | | | | | |
| CHX_Gel vs Doxy | - | - | - | - | - | - | NSPT vs CHX_Gel (moderate) | NSPT vs Doxy (high) | Moderate | 0 | - | -2 | Very low |
| CHX_Gel vs Mino | - | - | - | - | - | - | NSPT vs CHX_Gel (moderate) | NSPT vs Mino (moderate) | Moderate | 0 | - | -2 | Very low |
| CHX_Gel vs Tetra_Fyber | - | - | - | - | - | - | NSPT vs CHX_Gel (moderate) | NSPT vs Tetra_Fyber (moderate) | Moderate | 0 | - | -2 | Very low |
| CHX_Gel vs Tetra_Oint | - | - | - | - | - | - | NSPT vs CHX_Gel (moderate) | NSPT vs Tetra_Oint (moderate) | Moderate | 0 | - | -2 | Very low |
| Doxy vs Mino | - | - | - | - | - | - | NSPT vs Doxy (high) | NSPT vs Mino (moderate) | Moderate | 0 | - | -2 | Very low |
| Doxy vs Tetra_Fyber | - | - | - | - | - | - | NSPT vs Doxy (high) | NSPT vs Tetra_Fyber (moderate) | Moderate | 0 | - | -2 | Very low |
| Doxy vs Tetra_Oint | - | - | - | - | - | - | NSPT vs Doxy (high) | NSPT vs Tetra_Oint (moderate) | Moderate | 0 | - | -2 | Very low |
| Mino vs Tetra_Fyber | - | - | - | - | - | - | NSPT vs Mino (moderate) | NSPT vs Tetra_Fyber (moderate) | Moderate | 0 | - | -2 | Very low |
| Mino vs Tetra_Oint | - | - | - | - | - | - | NSPT vs Mino (moderate) | NSPT vs Tetra_Oint (moderate) | Moderate | 0 | - | -2 | Very low |
| Tetra_Fyber vs Tetra_Oint | - | - | - | - | - | - | NSPT vs Tetra_Fyber (moderate) | NSPT vs Tetra_Oint (moderate) | Moderate | 0 | - | -2 | Very low |

**GRADE Approach result for HbA1c at 6 months**

Criteria used to assess certainty of evidence was based on the Cochrane Training - [https://training.cochrane.org/resource/grade-approach-rate-certainty-evidence-network-meta-analysis-and-summary-findings-tables](about:blank) and apropos literature (listed below).

| **Criteria** | **Rated down direct estimate if:** | **Analysis** |
| --- | --- | --- |
| Risk of bias | When at least one study on the direct comparison presented high risk or some concerns due to problems in the randomization process and lack of blinding. | We rated down the certainty in one level due to some concerns and two levels due to high risk of bias in the randomization process.  On the other hand, since glycate hemoglobin is assessed through an automatic laboratory process, no problems with blinding were considered and we did not rate down the certainty. |
| Inconsistency | - If effect estimates were similar across studies (Guyatt et al. 2011b); - Overlap of 95%Cl (Guyatt et al. 2011b); - I^2^ for direct comparisons if was either moderate (30-60%), substantial (50-90%) or considerable (75%-100%) (Schunemann et al., 2021). - If only one study contributed to the direct effect estimate, the certainty was not rated down (Guyatt et al. 2011b). | We did not rate down the certainty for single studies forming the effect estimate.  One comparison had 2 studies included (CHX_Gel vs NSPT – Faramarzi et al., 2017 and Santos et al., 2013) and we downgraded one level of certainty of evidence because the confidence intervals overlapped, but the effect estimates were opposite. |
| Indirectness | Rate down if more than 30% of weight of pooled estimate came from studies which the populations had different characteristics (age, healthy condition) compared to the population of the PICO question (Brignardello-Petersen et al. 2018a). | We assessed indirectness for each comparison. For almost all comparisons, patients had similar characteristics regarding age (late adulthood/elders), T2D and the presence of periodontitis. All patients met the periodontitis case-definition, according to the New Periodontal Diseases Classification (Tonetti et al., 2018). Thus, there was no problems regarding periodontitis condition.  The initial glycated hemoglobin (HbA1c) was similar in all populations at the beginning of the study (ranging from 7.1 to 10.4%). The included patients had moderate to unsatisfactory metabolic control. |
| Publication bias | If industry funding was associated with a positive outcome favoring the sponsored treatment (Martins et al. 2019). | Of the studies included in this network meta-analysis,  only 1 received funded by the industry (DOXY vs NSPT – Lecio et al., 2019). However, although the results favored the treated group, there was no statistical difference for HbA1c in the study. Thus, the level was not downgraded due to publication bias. |
| **Criteria** | **Rated down indirect estimate if:** | **Analysis** |
| Intransitivity | If there were differences in study characteristics that may modify treatment effect in the direct comparisons that form the basis for the indirect estimate; and if those differences were regarding population, intervention, comparison, or outcome (Puhan et al. 2014). | For intransitivity, we considered the most dominant first order loop, or, in its absence, the closest direct comparison contributing to the effect estimate. Whenever the comparisons contributing to indirect evidence showed important modifications on population or glycated hemoglobin, one point was downgraded due to intransitivity. |
| **Criteria** | **Rated down NMA estimate if:** | **Analysis** |
| Incoherence | If node splitting test showed p-value <0.05, and direct, indirect and NMA evidence agreed with each other. (Brignardello-Petersen et al. 2018a). | Node split test was not feasible, due to the poorly connected network. In addition, it was not possible to compare the coherence between the direct and indirect estimates of the network meta-analysis. Thus, the certainty of evidence was not downgraded due to incoherence. |
| Imprecision | A partially contextualized approach was used, considering the large effect threshold according to Cohen’s classification (>0.8 SD) (Guyatt et al. 2011a; Schünemann et al. 2021; Brignardello-Petersen et al. 2021). | We downgraded in one level if the effect estimate crossed the minimum important difference (MID), considered as 0.5%; two levels if the confidence interval crossed the MID and the null effect line.  In the cases when the threshold was not crossed, the effect size was evaluated. OIS calculations were performed to large unplausible effects. Whenever OIS was not achieved, one level was downgraded (Brignardello-Petersen et al. 2021). |

**Table S2: Assessment of GRADE for direct and indirect comparisons evidence certainty for HbA1c (6 months comparisons).**

| **Comparison** | **Number of**  **Studies** | **Risk of Bias** | **Inconsistency** | **Indirectness** | **Publication Bias** | **Direct comparison Certainty** | **Indirect evidence comparison from the closest loop** | **Indirect evidence comparison from the closest loop** | **Lowest between both** | **Intransitivity** | **Incoherence for NMA** | **Imprecision for NMA** | **Final Certainty** |
| --- | --- | --- | --- | --- | --- | --- | --- | --- | --- | --- | --- | --- | --- |
| **DIRECT COMPARISONS** | | | | | | | | | | | | | |
| CHX_Gel vs NSPT | 2 | -1 (some conc) | -1 | 0 | 0 | Low | - | - | - | - | - | -2 | Very low |
| Doxy vs NSPT | 1 | 0 | 0 | 0 | 0 | High | - | - | - | - | - | -2 | Low |
| Mino vs NSPT | 1 | 0 | 0 | 0 | 0 | High | - | - | - | - | - | -2 | Low |
| **INDIRECT ONLY COMPARISONS** | | | | | | | | | | | | | |
| CHX_Gel vs Doxy | - | - | - | - | - | - | NSPT vs CHX_Gel (low) | NSPT vs Doxy (high) | Low | 0 | - | -2 | Very low |
| CHX_Gel vs Mino | - | - | - | - | - | - | NSPT vs CHX_Gel (low) | NSPT vs Mino (high) | Low | 0 | - | -2 | Very low |
| Doxy vs Mino | - | - | - | - | - | - | NSPT vs Doxy (high) | NSPT vs Mino (high) | High | 0 | - | -2 | Low |

**GRADE Approach result for PPD at 3 months**

Criteria used to assess certainty of evidence was based on the Cochrane Training - [https://training.cochrane.org/resource/grade-approach-rate-certainty-evidence-network-meta-analysis-and-summary-findings-tables](about:blank) and apropos literature (listed below).

| **Criteria** | **Rated down direct estimate if:** | **Analysis** |
| --- | --- | --- |
| Risk of bias | When at least one study on the direct comparison presented high risk or some concerns due to problems in the randomization process and lack of blinding. | We rated down the certainty in one level due to some concerns and two levels due to high risk of bias in the randomization process. One additional level was downgraded due to lack of outcome assessor blinding. |
| Inconsistency | - If effect estimates were similar across studies (Guyatt et al. 2011b); - Overlap of 95%Cl (Guyatt et al. 2011b); - I^2^ for direct comparisons if was either moderate (30-60%), substantial (50-90%) or considerable (75%-100%) (Schunemann et al., 2021). - If only one study contributed to the direct effect estimate, the certainty was not rated down (Guyatt et al. 2011b). | We did not rate down the certainty for single studies forming the effect estimate. One pair wise meta-analysis included two studies (CHX_Gel vs NSPT - Faramarzi et al., 2017 and Santos et al., 2013). Since the effect estimates were opposite and the confidence intervals did not completely overlap, one level was downgraded. |
| Indirectness | Rate down if more than 30% of weight of pooled estimate came from studies which the populations had different characteristics (age, healthy condition) compared to the population of the PICO question (Brignardello-Petersen et al. 2018a). | We assessed indirectness for each comparison. For almost all comparisons, patients had similar characteristics regarding age (late adulthood/elders), T2D and the presence of periodontitis. All patients met the periodontitis case-definition, according to the New Periodontal Diseases Classification (Tonetti et al., 2018). Thus, there was no problems regarding periodontitis condition.  The initial glycated hemoglobin (HbA1c) was similar in all populations at the beginning of the study (ranging from 7.1 to 10.4%). The included patients had moderate to unsatisfactory metabolic control. |
| Publication bias | If industry funding was associated with a positive outcome favoring the sponsored treatment (Martins et al. 2019). | Of the studies included in this network meta-analysis, 4 were funded by the industry. Of these, 2 showed clear favoring for the substance tested (CLM vs NSPT – Bajaj et al., 2012 and STZ vs NSPT – Priyanka et al., 2015). For these, the level of certainty of evidence was one level downgraded.  For comparisons AZT vs NSPT – Agarwal et al., 2012 and DOXY vs NSPT – Lecio et al., 2019, although the results was favorable for the test groups, there was no statistical difference between the groups. So, the level of certainty of evidence was not downgraded. |
| **Criteria** | **Rated down indirect estimate if:** | **Analysis** |
| Intransitivity | If there were differences in study characteristics that may modify treatment effect in the direct comparisons that form the basis for the indirect estimate; and if those differences were regarding population, intervention, comparison, or outcome (Puhan et al. 2014). | For intransitivity, we considered the most dominant first order loop, or, in its absence, the closest direct comparison contributing to the effect estimate. Whenever the comparisons contributing to indirect evidence showed important modifications on population or glycated hemoglobin, one point was downgraded due to intransitivity. |
| **Criteria** | **Rated down NMA estimate if:** | **Analysis** |
| Incoherence | If node splitting test showed p-value <0.05, and direct, indirect and NMA evidence agreed with each other. (Brignardello-Petersen et al. 2018a). | Node split test was not feasible, due to the poorly connected network. In addition, it was not possible to compare the coherence between the direct and indirect estimates of the network meta-analysis. Thus, the certainty of evidence was not downgraded due to incoherence. |
| Imprecision | A partially contextualized approach was used, considering the large effect threshold according to Cohen’s classification (>0.8 SD) (Guyatt et al. 2011a; Schünemann et al. 2021; Brignardello-Petersen et al. 2021). | We downgraded in one level if the effect estimate crossed the minimum important difference (MID), considered as 1mm; two levels if the confidence interval crossed the MID and the null effect line.  In the cases when the threshold was not crossed, the effect size was evaluated. OIS calculations were performed to large unplausible effects. Whenever OIS was not achieved, one level was downgraded (Brignardello-Petersen et al. 2021). |

**Table S3: Assessment of GRADE for direct and indirect comparisons evidence certainty for PPD (3 months comparisons).**

| **Comparison** | **Number of**  **Studies** | **Risk of Bias** | **Inconsistency** | **Indirectness** | **Publication Bias** | **Direct comparison Certainty** | **Indirect evidence comparison from the closest loop** | **Indirect evidence comparison from the closest loop** | **Lowest between both** | **Intransitivity** | **Incoherence for NMA** | **Imprecision for NMA** | **Final Certainty** |
| --- | --- | --- | --- | --- | --- | --- | --- | --- | --- | --- | --- | --- | --- |
| **DIRECT COMPARISONS** | | | | | | | | | | | | | |
| AZT vs NSPT | 1 | 0 | 0 | 0 | 0 | High | - | - | - | - | - | -2 | Low |
| CHX_Gel vs NSPT | 2 | -1 (some conc) | -1 | 0 | 0 | Low | - | - | - | - | - | -1 | Very low |
| CLM vs NSPT | 1 | 0 | 0 | 0 | -1 | Moderate | - | - | - | - | - | -1 | Low |
| Doxy vs NSPT | 1 | 0 | 0 | 0 | 0 | High | - | - | - | - | - | -1 | Moderate |
| Mino vs NSPT | 1 | -2 (high*) | 0 | 0 | 0 | Low | - | - | - | - | - | -2 | Very low |
| STZ vs NSPT | 1 | 0 | 0 | 0 | -1 | Moderate | - | - | - | - | - | -1 | Low |
| Tetra_Fiber vs NSPT | 1 | -2 (high*) | 0 | 0 | 0 | Low | - | - | - | - | - | -1 | Very low |
| **INDIRECT ONLY COMPARISONS** | | | | | | | | | | | | | |
| AZT vs CHX_Gel | - | - | - | - | - | - | NSPT vs AZT (high) | NSPT vs CHX_Gel (low) | Low | 0 | - | -2 | Very low |
| AZT vs CLM | - | - | - | - | - | - | NSPT vs AZT (high) | NSPT vs CLM (moderate) | Moderate | 0 | - | -2 | Very low |
| AZT vs Doxy | - | - | - | - | - | - | NSPT vs AZT (high) | NSPT vs Doxy (high) | High | 0 | - | -2 | Low |
| AZT vs Mino | - | - | - | - | - | - | NSPT vs AZT (high) | NSPT vs Mino (low) | Low | 0 | - | -2 | Very low |
| AZT vs STZ | - | - | - | - | - | - | NSPT vs AZT (high) | NSPT vs STZ (moderate) | Moderate | 0 | - | -2 | Very low |
| AZT vs Tetra_Fiber | - | - | - | - | - | - | NSPT vs AZT (high) | NSPT vs Tetra_Fiber (low) | Low | 0 | - | -2 | Very low |
| CHX_Gel vs CLM | - | - | - | - | - | - | NSPT vs CHX_Gel (low) | NSPT vs CLM (moderate) | Low | 0 | - | -1 | Very low |
| CHX_Gel vs Doxy | - | - | - | - | - | - | NSPT vs CHX_Gel (low) | NSPT vs Doxy (high) | Low | 0 | - | -1 | Very low |
| CHX_Gel vs Mino | - | - | - | - | - | - | NSPT vs CHX_Gel (low) | NSPT vs Mino (low) | Low | 0 | - | -2 | Very low |
| CHX_Gel vs STZ | - | - | - | - | - | - | NSPT vs CHX_Gel (low) | NSPT vs STZ (moderate) | Low | 0 | - | -1 | Very low |
| CHX_Gel vs Tetra_Fiber | - | - | - | - | - | - | NSPT vs CHX_Gel (low) | NSPT vs Tetra_Fiber (low) | Low | 0 | - | -1 | Very low |
| CLM vs Doxy | - | - | - | - | - | - | NSPT vs CLM (moderate) | NSPT vs Doxy (high) | Moderate | 0 | - | -1 | Low |
| CLM vs Mino | - | - | - | - | - | - | NSPT vs CLM (moderate) | NSPT vs Mino (low) | Low | 0 | - | -1 | Very low |
| CLM vs STZ | - | - | - | - | - | - | NSPT vs CLM (moderate) | NSPT vs STZ (moderate) | Moderate | 0 | - | -2 | Very low |
| CLM vs Tetra_Fiber | - | - | - | - | - | - | NSPT vs CLM (moderate) | NSPT vs Tetra_Fiber (low) | Low | 0 | - | -2 | Very low |
| Doxy vs Mino | - | - | - | - | - | - | NSPT vs Doxy (high) | NSPT vs Mino (low) | Low | 0 | - | -2 | Very low |
| Doxy vs STZ | - | - | - | - | - | - | NSPT vs Doxy (high) | NSPT vs STZ (moderate) | Moderate | 0 | - | -1 | Low |
| Doxy vs Tetra_Fiber | - | - | - | - | - | - | NSPT vs Doxy (high) | NSPT vs Tetra_Fiber (low) | Low | 0 | - | -1 | Very low |
| Mino vs STZ | - | - | - | - | - | - | NSPT vs Mino (low) | NSPT vs STZ (moderate) | Low | 0 | - | -1 | Very low |
| Mino vs Tetra_Fiber | - | - | - | - | - | - | NSPT vs Mino (low) | NSPT vs Tetra_Fiber (low) | Low | 0 | - | -2 | Very low |
| STZ vs Tetra_Fiber | - | - | - | - | - | - | NSPT vs STZ (moderate) | NSPT vs Tetra_Fiber (low) | Low | 0 | - | -2 | Very low |

*This comparison presented some concerns regarding randomization process and outcome assessment.

**GRADE Approach result for PPD at 6 months**

Criteria used to assess certainty of evidence was based on the Cochrane Training - [https://training.cochrane.org/resource/grade-approach-rate-certainty-evidence-network-meta-analysis-and-summary-findings-tables](about:blank) and apropos literature (listed below).

| **Criteria** | **Rated down direct estimate if:** | **Analysis** |
| --- | --- | --- |
| Risk of bias | When at least one study on the direct comparison presented high risk or some concerns due to problems in the randomization process and lack of blinding. | We rated down the certainty in one level due to some concerns and two levels due to high risk of bias in the randomization process. One additional level was downgraded due to lack of outcome assessor blinding. |
| Inconsistency | - If effect estimates were similar across studies (Guyatt et al. 2011b); - Overlap of 95%Cl (Guyatt et al. 2011b); - I^2^ for direct comparisons if was either moderate (30-60%), substantial (50-90%) or considerable (75%-100%) (Schunemann et al., 2021). - If only one study contributed to the direct effect estimate, the certainty was not rated down (Guyatt et al. 2011b). | We did not rate down the certainty for single studies forming the effect estimate. One pair wise meta-analysis included two studies (CHX_Gel vs NSPT - Faramarzi et al., 2017 and Santos et al., 2013). Since the effect estimates were opposite and the confidence intervals did not completely overlap, one level was downgraded. |
| Indirectness | Rate down if more than 30% of weight of pooled estimate came from studies which the populations had different characteristics (age, healthy condition) compared to the population of the PICO question (Brignardello-Petersen et al. 2018a). | We assessed indirectness for each comparison. For almost all comparisons, patients had similar characteristics regarding age (late adulthood/elders), T2D and the presence of periodontitis. All patients met the periodontitis case-definition, according to the New Periodontal Diseases Classification (Tonetti et al., 2018). Thus, there was no problems regarding periodontitis condition.  The initial glycated hemoglobin (HbA1c) was similar in all populations at the beginning of the study (ranging from 7.1 to 10.4%). The included patients had moderate to unsatisfactory metabolic control. |
| Publication bias | If industry funding was associated with a positive outcome favoring the sponsored treatment (Martins et al. 2019). | Of the studies included in this network meta-analysis, 4 were funded by the industry. Of these, 2 showed clear favoring for the substance tested (CLM vs NSPT – Bajaj et al., 2012 and STZ vs NSPT – Priyanka et al., 2015). For these, the level of certainty of evidence was one level downgraded.  For comparisons AZT vs NSPT – Agarwal et al., 2012 and DOXY vs NSPT – Lecio et al., 2019, although the results was favorable for the test groups, there was no statistical difference between the groups. So, the level of certainty of evidence was not downgraded. |
| **Criteria** | **Rated down indirect estimate if:** | **Analysis** |
| Intransitivity | If there were differences in study characteristics that may modify treatment effect in the direct comparisons that form the basis for the indirect estimate; and if those differences were regarding population, intervention, comparison, or outcome (Puhan et al. 2014). | For intransitivity, we considered the most dominant first order loop, or, in its absence, the closest direct comparison contributing to the effect estimate. Whenever the comparisons contributing to indirect evidence showed important modifications on population or glycated hemoglobin, one point was downgraded due to intransitivity. |
| **Criteria** | **Rated down NMA estimate if:** | **Analysis** |
| Incoherence | If node splitting test showed p-value <0.05, and direct, indirect and NMA evidence agreed with each other. (Brignardello-Petersen et al. 2018a). | Node split test was not feasible, due to the poorly connected network. In addition, it was not possible to compare the coherence between the direct and indirect estimates of the network meta-analysis. Thus, the certainty of evidence was not downgraded due to incoherence. |
| Imprecision | A partially contextualized approach was used, considering the large effect threshold according to Cohen’s classification (>0.8 SD) (Guyatt et al. 2011a; Schünemann et al. 2021; Brignardello-Petersen et al. 2021). | We downgraded in one level if the effect estimate crossed the minimum important difference (MID), considered as 1mm; two levels if the confidence interval crossed the MID and the null effect line.  In the cases when the threshold was not crossed, the effect size was evaluated. OIS calculations were performed to large unplausible effects. Whenever OIS was not achieved, one level was downgraded (Brignardello-Petersen et al. 2021). |

**Table S4: Assessment of GRADE for direct and indirect comparisons evidence certainty for PPD (6 months comparisons).**

| **Comparison** | **Number of**  **Studies** | **Risk of Bias** | **Inconsistency** | **Indirectness** | **Publication Bias** | **Direct comparison Certainty** | **Indirect evidence comparison from the closest loop** | **Indirect evidence comparison from the closest loop** | **Lowest between both** | **Intransitivity** | **Incoherence for NMA** | **Imprecision for NMA** | **Final Certainty** |
| --- | --- | --- | --- | --- | --- | --- | --- | --- | --- | --- | --- | --- | --- |
| **DIRECT COMPARISONS** | | | | | | | | | | | | | |
| AZT vs NSPT | 1 | 0 | 0 | 0 | 0 | High | - | - | - | - | - | -2 | Low |
| CHX_Gel vs NSPT | 2 | -1 (some conc) | -1 | 0 | 0 | Low | - | - | - | - | - | -1 | Very low |
| CLM vs NSPT | 1 | 0 | 0 | 0 | -1 | Moderate | - | - | - | - | - | -1 | Low |
| Doxy vs NSPT | 1 | 0 | 0 | 0 | 0 | High | - | - | - | - | - | -1 | Moderate |
| Mino vs NSPT | 1 | -2 (high*) | 0 | 0 | 0 | Low | - | - | - | - | - | -1 | Very low |
| STZ vs NSPT | 1 | 0 | 0 | 0 | -1 | Moderate | - | - | - | - | - | 0 | Moderate |
| **INDIRECT ONLY COMPARISONS** | | | | | | | | | | | | | |
| AZT vs CHX_Gel | - | - | - | - | - | - | NSPT vs AZT (high) | NSPT vs CHX_Gel (low) | Low | - | - | -1 | Very low |
| AZT vs CLM | - | - | - | - | - | - | NSPT vs AZT (high) | NSPT vs CLM (moderate) | Moderate | - | - | -2 | Very low |
| AZT vs Doxy | - | - | - | - | - | - | NSPT vs AZT (high) | NSPT vs Doxy (high) | High | - | - | -2 | Low |
| AZT vs Mino | - | - | - | - | - | - | NSPT vs AZT (high) | NSPT vs Mino (low) | Low | - | - | -2 | Very low |
| AZT vs STZ | - | - | - | - | - | - | NSPT vs AZT (high) | NSPT vs STZ (moderate) | Moderate | - | - | 0 | Moderate |
| CHX_Gel vs CLM | - | - | - | - | - | - | NSPT vs CHX_Gel (low) | NSPT vs CLM (moderate) | Low | - | - | -1 | Very low |
| CHX_Gel vs Doxy | - | - | - | - | - | - | NSPT vs CHX_Gel (low) | NSPT vs Doxy (high) | Low | - | - | -1 | Very low |
| CHX_Gel vs Mino | - | - | - | - | - | - | NSPT vs CHX_Gel (low) | NSPT vs Mino (low) | Low | - | - | -2 | Very low |
| CHX_Gel vs STZ | - | - | - | - | - | - | NSPT vs CHX_Gel (low) | NSPT vs STZ (moderate) | Low | - | - | 0 | Low |
| CLM vs Doxy | - | - | - | - | - | - | NSPT vs CLM (moderate) | NSPT vs Doxy (high) | Moderate | - | - | -1 | Low |
| CLM vs Mino | - | - | - | - | - | - | NSPT vs CLM (moderate) | NSPT vs Mino (low) | Low | - | - | -2 | Very low |
| CLM vs STZ | - | - | - | - | - | - | NSPT vs CLM (moderate) | NSPT vs STZ (moderate) | Moderate | - | - | -1 | Low |
| Doxy vs Mino | - | - | - | - | - | - | NSPT vs Doxy (high) | NSPT vs Mino (low) | Low | - | - | -2 | Very low |
| Doxy vs STZ | - | - | - | - | - | - | NSPT vs Doxy (high) | NSPT vs STZ (moderate) | Moderate | - | - | 0 | Moderate |
| Mino vs STZ | - | - | - | - | - | - | NSPT vs Mino (low) | NSPT vs STZ (moderate) | Low | - | - | 0 | Low |

*This comparison presented some concerns regarding outcome assessment.

**Supplement References**

Brignardello-Petersen R, Bonner A, Alexander PE, Siemieniuk RA, Furukawa TA, Rochwerg B, Hazlewood GS, Alhazzani W, Mustafa RA, Murad MH, Puhan MA, Schünemann HJ, Guyatt GH; GRADE Working Group. Advances in the GRADE approach to rate the certainty in estimates from a network meta-analysis. J Clin Epidemiol. 2018 Jan;93:36-44. doi: 10.1016/j.jclinepi.2017.10.005. (a)

Brignardello-Petersen R, Murad MH, Walter SD, McLeod S, Carrasco-Labra A, Rochwerg B, Schünemann HJ, Tomlinson G, Guyatt GH; GRADE Working Group. GRADE approach to rate the certainty from a network meta-analysis: avoiding spurious judgments of imprecision in sparse networks. J Clin Epidemiol. 2019 Jan;105:60-67. Doi: 10.1016/j.jclinepi.2018.08.022.

Brignardello-Petersen R, Mustafa RA, Siemieniuk RAC, Murad MH, Agoritsas T, Izcovich A, Schünemann HJ, Guyatt GH; GRADE Working Group. GRADE approach to rate the certainty from a network meta-analysis: addressing incoherence. J Clin Epidemiol. 2019 Apr;108:77-85. Doi: 10.1016/j.jclinepi.2018.11.025. (b)

Brignardello-Petersen R, Guyatt GH, Mustafa RA, Chu DK, Hultcrantz M, Schünemann HJ, Tomlinson G. GRADE guidelines 33: Addressing imprecision in a network meta-analysis. J Clin Epidemiol. 2021 Nov;139:49-56. doi: 10.1016/j.jclinepi.2021.07.011. Epub 2021 Jul 19.

Guyatt GH, Oxman AD, Kunz R, Brozek J, Alonso-Coello P, Rind D, Devereaux PJ, Montori VM, Freyschussi B, Vist G et al. 2011a. Grade guidelines 6. Rating the quality of evidence -imprecision. J Clin Epidemiol. 64:1283-1293. (b)

Guyatt GH, Oxman AD, Kunz R, Woodcock J, Brozek J, Helfand M, Alonso-Coello P, Glasziou P, Jaeschke R, Akl EA et al. 2011b. Grade guidelines: 7. Rating the quality of evidence--inconsistency. J Clin Epidemiol. 64(12):1294-1302. (a)

Martins CC, Riva JJ, Firmino RT, Colunga-Lozano LE, Granville-Garcia AF, Zhang Y, Schünemann HJ. 2019. Conflict of interest is not associated with positive conclusions in toothpaste trials: A systematic survey. J Clin Epidemiol. 108:140-146.

Puhan MA, Schünemann HJ, Murad MH, Li T, Brignardello-Petersen R, Singh JA, Kessels AG, Guyatt GH; GRADE Working Group. A GRADE Working Group approach for rating the quality of treatment effect estimates from network meta-analysis. BMJ. 2014 Sep 24;349:g5630. doi: 10.1136/bmj.g5630.

Schünemann HJ, Vist GE, Higgins JPT, Santesso N, Deeks JJ, Glasziou P, Akl EA, Guyatt GH. Chapter 15: Interpreting results and drawing conclusions. In: Higgins JPT, Thomas J, Chandler J, Cumpston M, Li T, Page MJ, Welch VA (editors). Cochrane Handbook for Systematic Reviews of Interventions version 6.2 (updated February 2021). Cochrane, 2021. Available from [www.training.cochrane.org/handbook](about:blank).

Tonetti MS, Greenwell H, Kornman KS. Staging and grading of periodontitis: Framework and proposal of a new classification and case definition. J Periodontol. 2018;89(Suppl 1):S159-S172. doi:10.1002/JPER.18-0006
