# Supplementary material for: Efficacy of Adjunctive Local Antimicrobials to Non-Surgical Periodontal Therapy in Pocket Reduction and Glycemic Control of Patients with Type 2 Diabetes: A Network Meta-Analysis
Source: Curr Diabetes Rev. 2024 Sep 3;21(7):E15733998320667. doi: 10.2174/0115733998320667240805045742 (PMC12082567; doi:10.2174/0115733998320667240805045742)
Supplement: Supplementary file 1 [file CDR-21-7-E15733998320667_SD1.zip › CDR-21-7-E15733998320667_SD1/S2 - Excluded articles and reasons for exclusion.doc]

**Supplementary file 2 - Excluded articles and reason for exclusion. (n=20).**

| **Author, year** | **Reason for exclusion** |
| --- | --- |
| Al-Ahmari. 2021 | 8 |
| Engebretson & Hey-Hadavi. 2011 | 8 |
| Grossi et al. 1997 | 8 |
| Haerian-Ardakani et al. 2014a | 5 |
| Haerian-Ardakani et al. 2014b | 11 |
| Hincapié et al. 2014 | 8 |
| Komatsu et al. 2022 | 8 |
| Miranda et al. 2014 | 8 |
| Peña Sisto et al. 2018 | 8 |
| Qureshi et al. 2021 | 8 |
| Torkzaban & Rabienejad. 2018 | 8 |
| CTRI/2019/02/017389 | 4 |
| CTRI/2019/07/020326 | 4 |
| CTRI/2021/05/033474 | 4 |
| IRCT2013092614774N1 | 4 |
| IRCT2015010517587N8 | 4 |
| IRCT2015010517587N9 | 4 |
| NCT02062047 | 4 |
| NCT02735837 | 4 |
| NCT06027151 | 4 |

1- Reviews, letters, personal opinions, books or book chapters, panel and conference abstracts (n=0), 2- Wrong design studies. (n=0), 3- Wrong sample studies. (n=0), 4- Study Protocols (n=9), 5- Studies written in non-Latin alphabets (n=1), 6- Wrong outcome (n=0), 7- Wrong population (n=0), 8- Wrong drug (systemic use or wrong vehicle) (n=9), 9-Confused effects (n=0), 10- No full text available (n=0), 11- duplicate study (n=1)

REFERENCES

1. Al-Ahmari MM. Effect of Antibiotics and Carbohydrate Diet Control Instructions in Improving Glycated Hemoglobin among Type II Diabetic Patients with Periodontitis. J Contemp Dent Pract. 2021;22(10):1118–1122.
2. Engebretson SP & Hey-Hadavi J. Sub-antimicrobial doxycycline for periodontitis reduces hemoglobin A1c in subjects with type 2 diabetes: a pilot study. Pharmacol Res. 2011 Dec;64(6):624-9. doi: 10.1016/j.phrs.2011.06.024.
3. Grossi SG, Skrepcinsk FB, DeCaro T, et al. Treatment of Periodontal Disease in Diabetics Reduces Glycated Hemoglobin. J Periodontol 1997;68(8):713-19.
4. Haerian-Ardakani A, Asadi Y, Afkhami M, et al. Effect of topical tetracycline gel with non surgical periodontal therapy on Hba1c and lipid profile in type 2 diabetic patients: a clinico-biochemical study. J Shahid Sadoughi Univ Med Sci 2014; 22(4):1387-95.
5. Haerian-Ardakani A, Asadi Y, Afkhami M, et al. Effect of topical tetracycline gel with non surgical periodontal therapy on Hba1c and lipid profile in type 2 diabetic patients. J Shahid Sadoughi Univ Med Sci 2014; 22(4):1387-95.
6. Hincapié JP, Castrillón CA, Yepes FL, et al. Microbiological effects of periodontal therapy plus azithromycin in patients with diabetes: results from a randomized clinical trial. Acta Odontol Latinoam. 2014;27(2):89-95. doi: 10.1590/S1852-48342014000200008.
7. Komatsu S, Oshikiri S, Nagano T, et al. Effects of One-Stage Full-Mouth Scaling and Root Planing with Azithromycin on Diabetes and Periodontal Disease: A Randomized Controlled Trial. Antibiotics. 2022;11,1266. doi: 10.3390/antibiotics11091266.
8. Miranda TS, Feres M, Perez-Chaparro PJ, et al. Metronidazole and amoxicillin as adjuncts to scaling and root planing for the treatment of type 2 diabetic subjects with periodontitis: 1-year outcomes of a randomized placebo-controlled clinical trial. J Clin Periodontol. 2014 Sep;41(9):890-9. doi: 10.1111/jcpe.12282.
9. Peña Sisto M, Silva MC, Avalo WS, et al. Efectividad del tratamiento periodontal en el control metabólico de pacientes con diabetes mellitus. MEDISAN. 2018;22(3):240-247.
10. Qureshi A., Bokhari SAH, Haque Z, et al. Clinical efficacy of scaling and root planing with and without metronidazole on glycemic control: three-arm randomized controlled trial. BMC Oral Health. 2021;21(1):253. doi: 10.1186/s12903-021-01620-1.
11. Torkzaban P & Rabienejad N. Effect of non-surgical periodontal therapy with and without 0.2% chlorhexidine rinse on metabolic control of type 2 diabetic patients. Avicenna J Dent Res. 2018;10(3):83-88. doi: 10.34172/ajdr.2018.18.
12. CTRI/2019/02/017389, To evaluate the effectiveness of chlorhexidine chip as an adjunct to scaling and root planing in patient with type 2 diabetes mellitus. https://trialsearch.who.int/Trial2.aspx?TrialID=CTRI/2019/02/017389 - Volume 0, Issue 0, pp. – published 2019-02-01.
13. CTRI/2019/07/020326, Comparison of glycated hemoglobin levels after cleaning of teeth with and without application of chlorhexidine metronidazole gel on gums in chronic periodontitis patient with type 2 diabetes mellitus. https://trialsearch.who.int/Trial2.aspx?TrialID=CTRI/2019/07/020326 - Volume 0, Issue 0, pp. – published 2019-07-23.
14. CTRI/2021/05/033474, Benefits of two locally delivered agents on cytokine level in diabetic patients with chronic periodontitis. https://trialsearch.who.int/Trial2.aspx?TrialID=CTRI/2021/05/033474 - Volume 0, Issue 0, pp. – published 2021-05-07.
15. IRCT2013092614774N1, Effects of SRP on blood glucose level reduction in diabetic patients.

https://trialsearch.who.int/Trial2.aspx?TrialID=IRCT2013092614774N1 - Volume 0, Issue 0, pp. - published 2014-02-06.

16. IRCT2015010517587N8, A randomized, placebo controlled trial The effect of non-surgical periodontal therapy with adjunctive topical doxycycline gel on HbA1c in patients with type 2 diabetes mellitus (DM). https://trialsearch.who.int/Trial2.aspx?TrialID=IRCT2015010517587N8 – Volume 0, Issue 0, pp. – published 2015-02-10.
17. IRCT2015010517587N9, A randomized double-blind placebo-controlled, clinical trial the effect of non-surgical periodontal therapy plus subgingivally azithromycin gel on HbA1c in patients with type 2 diabetes mellitus (DM). https://trialsearch.who.int/Trial2.aspx?TrialID=IRCT2015010517587N9 - Volume 0, Issue 0, pp. - published 2015-02-18.
18. NCT02062047, Full-mouth and Partial-mouth Scaling and Root Planing in Type 2 Diabetic Subjects. https://clinicaltrials.gov/show/NCT02062047 - Volume 0, Issue 0, pp. – published 2014-02-13.
19. NCT02735837, The Effect of Topical Doxycycline Gel on HbA1c in Patients With Type 2 Diabetes Mellitus (DM)®.  https://clinicaltrials.gov/show/NCT02735837 - Volume 0, Issue 0, pp. – published 2016-04-13.
20. NCT06027151, Comparing the Efficacy of Metronidazole and Minocycline Gels for the Treatment of Diabetic Periodontitis Patients. https://clinicaltrials.gov/ct2/show/NCT06027151 - Volume 0, Issue 0, pp. - published 2023-01-01.
